# Heparin affinity purification of extracellular vesicles

**DOI:** 10.1038/srep10266

**Published:** 2015-05-19

**Authors:** Leonora Balaj, Nadia A. Atai, Weilin Chen, Dakai Mu, Bakhos A. Tannous, Xandra O. Breakefield, Johan Skog, Casey A. Maguire

**Affiliations:** 1 Department of Neurology, Massachusetts General Hospital and Program in Neuroscience, Harvard Medical School, Boston, MA.; 2 Departments of Radiology, Massachusetts General Hospital and Program in Neuroscience, Harvard Medical School, Boston, MA.; 3 Department of Cell Biology and Histology, Academic Medical Center (AMC), University of Amsterdam, The Netherlands.; 4 Exosome Diagnostics Inc. Cambridge, MA.

## Abstract

Extracellular vesicles (EVs) are lipid membrane vesicles released by cells. They carry active biomolecules including DNA, RNA, and protein which can be transferred to recipient cells. Isolation and purification of EVs from culture cell media and biofluids is still a major challenge. The most widely used isolation method is ultracentrifugation (UC) which requires expensive equipment and only partially purifies EVs. Previously we have shown that heparin blocks EV uptake in cells, supporting a direct EV-heparin interaction. Here we show that EVs can be purified from cell culture media and human plasma using ultrafiltration (UF) followed by heparin-affinity beads. UF/heparin-purified EVs from cell culture displayed the EV marker Alix, contained a diverse RNA profile, had lower levels of protein contamination, and were functional at binding to and uptake into cells. RNA yield was similar for EVs isolated by UC. We were able to detect mRNAs in plasma samples with comparable levels to UC samples. In conclusion, we have discovered a simple, scalable, and effective method to purify EVs taking advantage of their heparin affinity.

Extracellular vesicles (EVs) have been increasingly recognized as carriers of messages in cell-to-cell communication and biomarkers for different diseases, as well as for gene and drug delivery^1^. These vesicles can be formed internally by initial invagination of the plasma membrane into endosomes, then in-budding of vesicles into endosomal-derived multivesicular bodies (MVBs) and later fusion of the MVBs with the plasma membrane to release vesicles into the intercellular surrounding^2^,^3^,^4^. EVs are also formed and released directly from the plasma membrane during cytoskeletal rearrangement, budding, or apoptosis^3^. Cancer cells may also release a subpopulation of retroviral-like particles which are likely generated upon increased transcription of endogenous retroviral sequences^5^,^6^.

Isolation and purification of released EVs remains a challenge. Methods currently used include differential and high speed UC^7^, separation on density gradients^8^, proprietary commercial kits, immune-affinity purification^9^,^10^ and microfluidics^11^. UC, in addition to requiring specialized and expensive equipment, allows sedimentation of different types of EVs, including large oncosomes^12^ and apoptotic bodies^3^,^13^ along with co-sedimentation of protein aggregates, such as BSA^14^, HDL^15^ and nucleic acids^16^. Furthermore, EVs tend to cluster together and form large aggregates in the pellet which are difficult to separate and may interfere with quantification and alter uptake of EVs by recipient cells^17^. Density gradients are lengthy and laborious with low yield, and may not be the best criteria to separate different types of EVs, as it may vary significantly between samples, especially in the case of cancer where the production and size of EVs increases^6^ , with differing contents from EVs released from normal cells^18^. Other methods do not allow large scale EV isolation and/or require cocktails of cell- or disease-specific antibodies as well as lengthy optimizations. Heparin is a highly-sulfated glycosaminoglycan with the highest negative charge density of any known biological molecule^19^ and is primarily produced by mast cells^20^.

Heparan sulfate proteoglycans (HSPG) are cell surface receptors which are structurally related to heparin^20^ and are important in a variety of biological processes^21^, with ligand binding to HSPG typically being blocked by incubating with a molar excess of heparin. We have previously shown that addition of heparin to labeled EVs derived from 293T cells almost entirely inhibited their uptake by unlabeled recipient 293T cells^22^; and recently we have shown that heparin blocks transfer of tumor cell EVs to recipient cells^23^. In addition, another group showed that tumor-derived EVs require HSPG to be on the recipient cell surface for uptake^24^. All of these data led to our hypothesis that heparin can directly bind to the surface of EVs.

We set out with the following two primary goals of using heparin affinity for EVs: (1) to isolate relatively pure, intact EVs from cell culture media to be used in functional biological assays; (2) to isolate EV-associated RNA from a biofluid to be used for biomarker analysis. Here we show that a heparin affinity matrix can be used to purify EVs from conditioned cell culture media, as well as from blood plasma. We characterized the protein and nucleic acid content, yield, morphology, and uptake dynamics of heparin purified cell culture-derived EVs and compared it to that of the standard method of purification, UC, as well as a commercially available EV isolation kit.

## Results

### Extracellular vesicles bind to heparin-conjugated agarose beads

**Figure 1:**
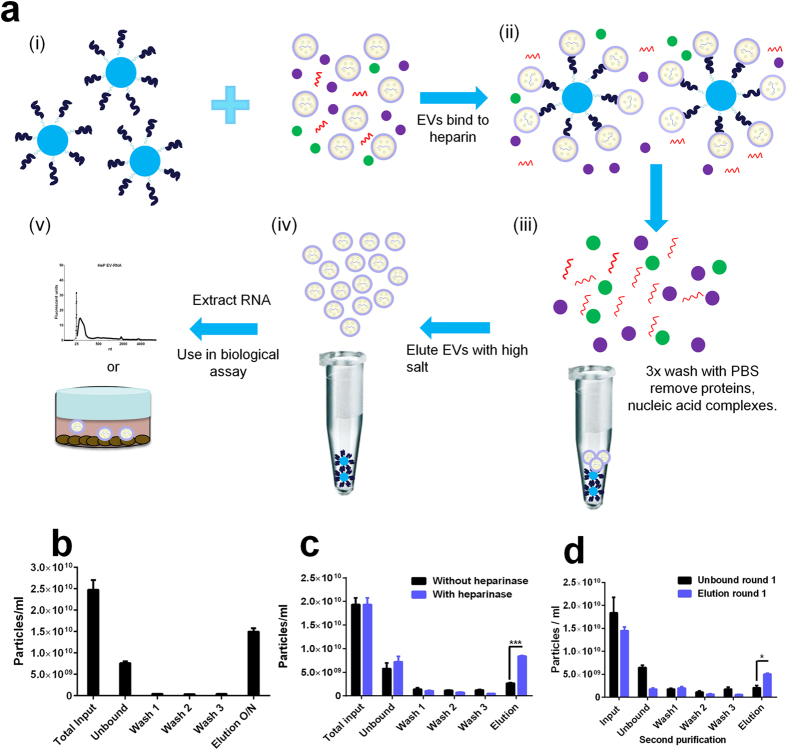
Extracellular vesicles are efficiently isolated and purified using heparin-coated agarose beads. (a) Heparin coated agarose beads are incubated with EVs released from a variety of cells lines, (i), to yield an EV/heparin complex, (ii). Free floating proteins and nucleic acids are washed away with PBS, (iii). Beads are the incubated overnight with 2.15 M NaCl and the EVs are released and collected by spinning down the beads and collecting the supernatant (iv). Collected EVs are used as a source of RNA (biomarker) or used in biological assays (v**). (b)** Nanoparticle tracking analysis (NTA) counts of heparin-purified human 293T-derived EVs eluted with 2.15 M NaCl overnight at 4 °C following 3 wash steps. (**c**) To show specific heparin affinity we incubated heparin beads overnight with EVs, then rinsed beads 3 times with PBS and treated with *Bacteroides Heparinase I* or incubation buffer without heparinase and fractions were analyzed by NTA. (**d**) EVs were mixed with heparin beads and one round of purification was performed. The unbound and eluted fractions from round one were separately incubated with a fresh batch of heparin beads and round 2 purification performed on these samples. NTA was performed on each fraction of round 2 purification.

Twenty ml of conditioned media from 293T cells was processed as described in methods and concentrated down to 1 ml using low speed centrifugation and a 100 kDa molecular weight cutoff ultrafiltration (UF) centrifugal device. The sample was mixed with 1 ml of prewashed heparin-coated agarose beads and incubated on a tube rotator at 4 °C overnight. Beads were washed three times with PBS and EVs were eluted with 2.15 M NaCl in PBS overnight at +4 °C (Fig. 1a). We used the established technology^14^,^25^,^26^ of Nanoparticle Tracking Analysis (NTA) to evaluate particle numbers in our conditioned cell culture media samples and observed 60% recovery of the input EVs (Fig. 1b). An additional 20% of particle counts was found in the unbound and wash fractions leaving approximately 20% unaccounted for. Some of this may be residual EVs which are still bound to the beads after elution or become damaged at some point. Before counting, samples were diluted to physiological levels of salt (~150 mM) to compensate for any EV shrinking in the high-salt buffer. We compared the NTA size profiles between heparin-purified and UC isolated EVs and found them to be similar in size distribution (Supplementary Fig. 1a). Comparison of the unbound and eluted EV fractions using heparin beads indicated a slightly higher diameter in size for the eluted EVs (Supplementary Fig. 1b). To determine whether the binding of EVs to the beads was heparin-specific, we mixed EVs with heparin beads overnight at 4 °C and then performed three washes with PBS as in Fig 1b. For elution, samples were either treated with control buffer or treated with heparinase to digest heparin thereby releasing EVs from the agarose beads (Fig. 1c). The samples digested with heparinase had a significantly higher yield of EVs (p ≤ 0.00001) compared to mock treated EV/heparin beads (no heparinase), as measured by NTA. The slight increase in the elution fraction of mock treated sample compared to the washes may be due to the incubation step at 30 °C in reaction buffer, required for heparinase to be active. Further evidence for a direct EV/heparin interaction was obtained by comparing mock-treated EVs or with EVs previously incubated with 0.1 mg/ml soluble heparin (Supplementary Fig. 2a). We found that binding of EVs to heparin beads in the presence of excess soluble heparin was significantly less efficient when compared to mock treated EVs (no soluble heparin), as there were significantly more unbound EVs in the sample pre-incubated with heparin than the mock treated sample (p ≤ 0.04). This block in binding resulted in 1.8-fold less EVs recovered in the elution step as expected (p ≤ 0.01). As a final control for nonspecific binding of EVs to the agarose bead support matrix we incubated an equal volume of 293T cell conditioned media with either the heparin agarose beads or agarose beads without conjugated heparin. Next we performed the purification process for both samples and analyzed all fractions by NTA. We found a 1.8-fold higher amount of particles in the unbound fraction of control beads alone, compared to heparin beads (p < 0.04; Supplementary Fig. 2b). In contrast, a 3.6-fold lower number of particles were present in the elution fraction of EVs incubated with the control beads alone, compared with heparin beads (p < 0.02). This result provides further evidence for a specific heparin/EV binding interaction.

We wondered if there may be distinct populations of EVs that bind heparin better than others. To test this hypothesis we incubated 293T-derived EVs with heparin beads and retained all fractions (unbound, washes, elutions). Salt was removed from the eluted fraction by UF. Next we incubated the unbound fraction (i.e. those that didn’t bind heparin beads on round 1 purification) or the eluted fraction (bound heparin) with a subsequent batch of heparin beads. For each sample, we performed washes and elutions, and particles were counted by NTA. Interestingly, the unbound fraction from purification round 1 gave 35% of unbound particles in round 2, while the round 1 eluted samples had only 12% in the unbound fraction in round 2 (p < 0.003; Fig. 1d). On the other hand, there was a 2.5-fold higher particle count in the round 2 elution from the round 1 eluted sample compared to round 2 elution with the round 1 unbound sample (p < 0.004; Fig. 1d). This result suggests that the 293T EVs are comprised of a mixed pool with some binding more strongly to heparin than others. We also show that EVs from U87 cells (Supplementary Fig. 3a) as well as HUVEC cells (Supplementary Fig. 3b) can be purified using heparin beads, as determined by NTA counts with 30% and 28% recovery, respectively, following overnight salt elution at 4 °C.

### Heparin-purified EVs contain EV-associated biomarkers and lower levels of a contaminating protein

**Figure 2:**
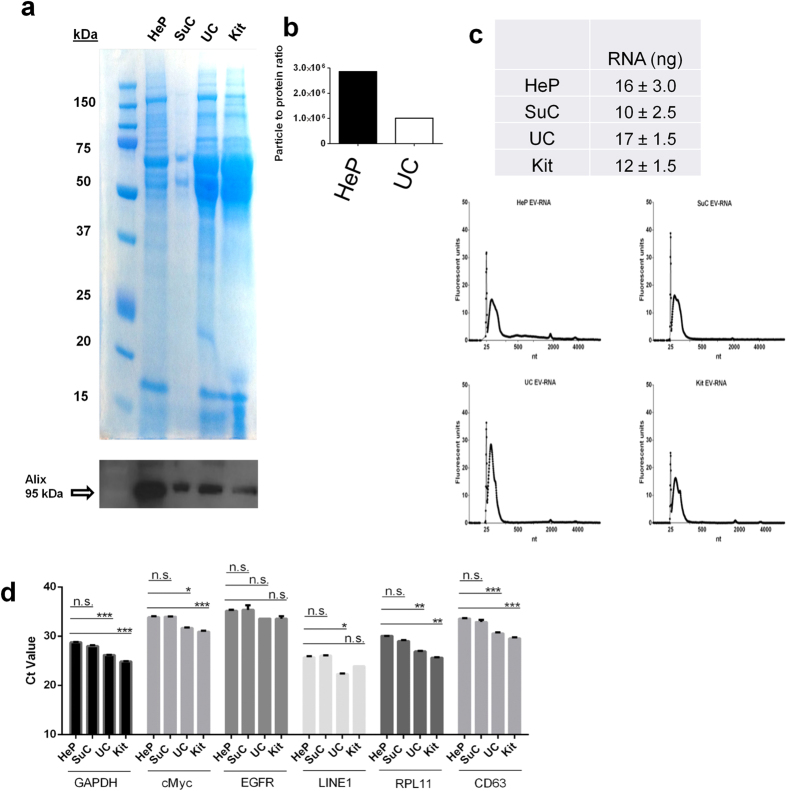
Characterization of total proteins and EV markers from heparin-purified (HeP), sucrose gradient-isolated (SuC) ultracentrifuged (UC), and commercial kit (kit)-isolated EVs . In two separate preparations from 293T cells, recovered EVs purified with each of these methods had EVs counted using NTA. (**a**) The EV number (2.1 × 10^10^ particles for each lane) was used to normalize protein loading on the SDS PAGE gel. Coomassie staining revealed EV associated and co-pelleting proteins in each sample. The samples were also probed by western blotting for the EV marker Alix, (a, bottom panel; band indicated with open arrow). (**b**) Particle to total protein ratio (**c**) RNA yields and bioanalyzer profiles extracted using the HeP, SuC, UC and the Kit methods. (**d**) The recovered RNA from each method was reverse transcribed into cDNA and used as input for the qRT-PCR. Levels of several mRNAs were determined. The data is represented as the average Ct values±s.d. (lower means higher levels of the mRNA sequence) and normalized to the housekeeping mRNA, GAPDH (n = 3). *p-*values were calculated using the two-tailed *t*-test (*p < 0.05, **p < 0.01, ***p < 0.001); n.s. = non-significant.

One ml of concentrated conditioned media (see Methods) was purified by heparin-coated beads (HeP), UC, sucrose gradient (SuC), or a commercial kit. Each sample was split into two fractions: fraction one was used for SDS-PAGE gel electrophoresis and Coomassie staining to visualize total protein associated with each purification method (Fig. 2a). Norm**a**lization was based on EV counts after purification. The second fraction was used for Western blot analysis of the EV-associated protein, Alix (Fig. 2a **bottom panel**). The strong bands in Fig. 2a for UC and kit-isolated EVs were between 50 and 75 kDa, with weaker bands in this size range seen for heparin-isolated and sucrose gradient purified EVs. As BSA (MW 69 kDa) has been reported to co-purify with EVs^14^, we also performed an anti-BSA immunoblot and found a broad, intense band around 69 kDa, detected in 293T UC EVs following SDS-gel electrophoresis and immunoblotting with an anti-BSA antibody, while this band was less intense in the heparin bead-purified sample (same number of EVs loaded; Supplementary Fig. 4). These BSA bands are likely the intense bands migrating between 50 and 75 kDa seen by Coomassie staining in Fig. 2a. Blotting for Alix revealed an intense band of the expected molecular weight for all samples (Fig. 2A, bottom). We determined the nanoparticle to protein ratio to estimate the purity of the preparations (see methods for calculation) and found heparin purified samples to have a 2.8-fold higher ratio (Fig. 2b). We repeated the SDS PAGE analysis of total proteins in 3 separate heparin bead purifications and compared it to UC (loading based on equal NTA particle counts, Supplementary Fig. 5a). Again, we observed much less protein in the heparin-purified samples compared to the UC sample. To validate our loading method, we also normalized sample loading based on nucleic acid, and as expected, the heparin purified sample had lower levels of total protein per lane (Supplementary Fig. 5b).

For RNA analyses, all samples were DNAse treated on column according to the manufacturer’s protocol. Heparin-purified EVs from 1 ml of concentrated conditioned media (starting volume 20 ml; 3.0 × 10^10^ particles/ml heparin beads) were eluted overnight in 1 ml of 2 M NaCl at 4 °C, ultracentrifuged at 100,000 x g for 90 minutes and lysed in 700 μl of Qiazol lysis buffer. We evaluated the RNA yields and profiles of the EV-RNA in heparin-purified samples and compared it to SuC, UC and kit isolated samples starting with the same EV numbers as determined by NTA (Fig. 2 c). We determined that EV-RNA yield was quite similar among the different purification methods (Fig. 2c). All methods displayed a peak in the small RNA region (25 – 200 nt) suggesting the presence of small RNAs/miRNA species, with smaller amounts of larger RNA species, such as ribosomal RNA peaks (Fig. 2c). Samples from the 4 different isolation methods were frozen at −80 °C and stored at +4 °C in the same fashion to account for RNA stability under different storage conditions. Next we performed qRT-PCR on the RNA (DNAse treated) isolated from these EV samples and showed that a variety of mRNA sequences (GAPDH, cMyc, EGFR, LINE1, RPL11, CD63) are readily detected within heparin-purified EVs similar to the other isolation methods (Fig. 2d). The levels of abundant cellular RNA species, such as GAPDH and RPL11, are significantly higher in UC and commercial kit samples indicating they may be present in vesicles, particles or aggregates that do not bind to heparin. Both these abundant messages were recovered at similar levels between sucrose gradients and heparin coated beads. Interestingly the EGFR and LINE1 mRNAs were similarly recovered among all different EV isolation methods. CD63 and cMyc mRNAs were recovered at slightly higher efficiency when purified using UC or the commercial kit. Overall the sucrose gradient and heparin purified EVs contained similar amounts of all mRNA sequences investigated (with the latter being a less laborious method and not requiring expensive laboratory equipment). No signal was detected in samples which did not include a reverse-transcriptase step, excluding the possibility of DNA contamination contributing to the Ct values detected (data not shown).

### Putative heparin-binding proteins on EVs

To attempt to identify EV-associated proteins in the heparin purified preparation which may be responsible for binding to the heparin matrix, we gel-extracted the intense ~65 and ~70 kDa bands from a Coomassie stained gel (Supplementary Fig. 6) and subjected them to mass spectrometry analysis (**Table I** and **II**). As expected, the most identified protein for the 70 kDa bands based on 40 unique peptides with ~50% coverage of the entire protein was bovine serum albumin (BSA), presumably a contaminant from fetal bovine serum in the cell culture media. For the 65 kDa sample, the most abundant reads were for bovine Alpha-2-HS-glycoprotein. Using the exosome database ExoCarta^27^, we searched the mass spectrometry hits to observe if the identified proteins had been previously reported to be associated with exosomes/EVs. We then analyzed which of the EV-associated proteins are known to be exposed on the cell/EV surface and searched if any putative heparin binding domains existed for those proteins (**Table I, II**). The second most abundant hit from the mass spectrometric analysis was Annexin A1 with 50% peptide coverage. Although the molecular weight of Annexin A1 is only 35 kDa, it is known to form dimers on SDS PAGE gels^28^,^29^, and may have migrated at the 70 kDa size. Annexin is reported to be on the vesicle surface^30^ and to bind to heparin^31^. Another major hit from the analysis was Zymogen granule protein 16 homolog B (ZG16p), also found in the ExoCarta database and shown to bind to heparin^32^. Other candidates found in the top 7 hits were the heat shock protein family members, human heat shock 70 kDa protein 1-like and human heat shock 70 kDa protein 1 A. These are also reported to be on the EV surface^33^,^34^ and bind to heparin^35^. Finally, histone H4 was detected in the mass spec results. Recently histones were reported to coat the EV surface and facilitate HSPG binding^36^.

### mRNA quantification in heparin purified and ultracentrifuged human plasma samples

**Figure 3:**
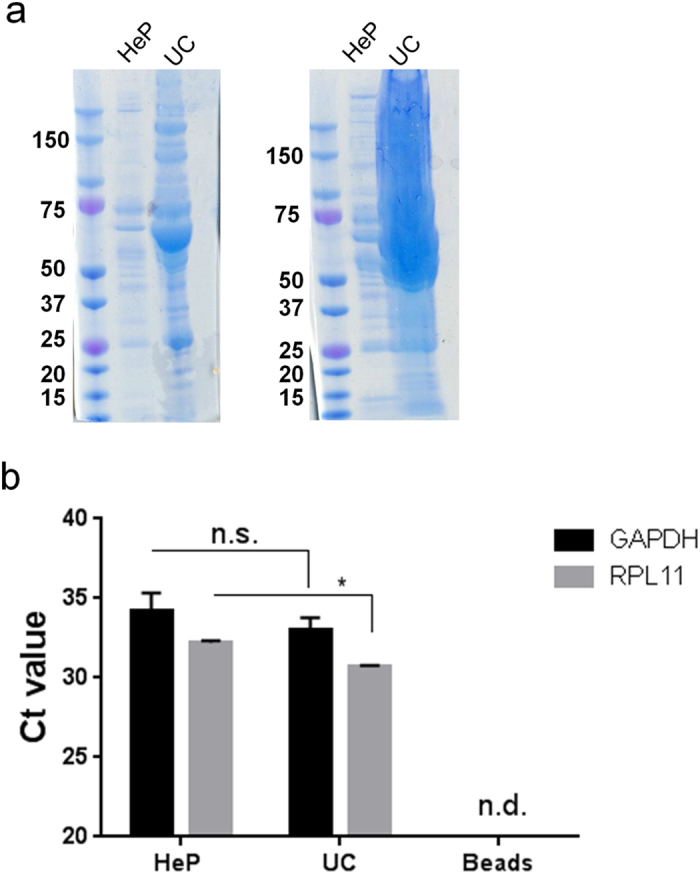
mRNA quantification of heparin-purified and ultracentrifuged human plasma- derived EVs . **(a)** Total protein in equal volumes from each isolation method. Left gel, 5 μl of sample loaded; right gel, 26 μl of sample loaded. **(b)** Two ml of plasma samples from healthy controls were thawed on ice, passed through a 100 kDa Amicon filter (Millipore) and filter-retained EVs washed with PBS buffer. The sample was split into three aliquots; one aliquot of washed EVs was added to biotin heparin - streptavidin coated magnetic beads and incubated on a rotator overnight at +4 °C to allow binding. A second aliquot was added to mock treated streptavidin coated magnetic beads. The third aliquot was stored at +4 °C until day 2 and then ultracentrifuged at 100,000 x g to collect EVs. RNA was extracted from all three samples using the miRNeasy kit (Qiagen) and analyzed for the presence of 2 mRNA messages: GAPDH and RPL11. Data is shown in Ct values (lower Ct means higher levels) and normalized to initial input (n.d. = not detected). Note: The mean and standard deviations are calculated from 3 healthy blood donor samples.

Plasma samples collected under approved IRB from healthy donors were stored at −80C until analysis. For each experiment, two ml of plasma from two healthy donors were washed three times with PBS using a nanofiltration device and divided in thirds, for heparin-affinity isolation, control beads, and UC. Biotinylated heparin bound to Streptavidin CI coated magnetic beads was used to isolate EVs from plasma samples. EVs were allowed to bind to heparin overnight at +4C. Magnetic beads were washed three times with PBS and lysed directly in RIPA buffer for SDS PAGE analysis of protein or Qiazol for RNA analysis by qRT-PCR. We loaded SDS PAGE gels with 5 μl or 26 μl of sample from each preparation of plasma EVs and visualized total protein by Coomassie blue. Similar to our results with cell culture media, heparin purified samples had greatly reduced protein compared to UC samples (Fig. 3a). We also detected HSP70 protein marker in the heparin isolated samples but not in the UC samples which may likely be due to co-isolation of many contaminants (Supplementary Fig. 7). GAPDH and RPL11 mRNAs were detected in heparin-bead isolated as well as UC samples (n.s. and p ≤ 0.03 respectively) (Fig. 3b). Both mRNAs had comparable bionalyzer profiles (Supplementary Fig. 8) in the heparin isolated and UC EVs. No transcripts were detected after 40 cycles when we attempted to isolated EVs with the Streptavidin beads alone (no heparin bound; n.d. = not detected; Fig. 3b).

### Electron microscopic analysis of isolated extracellular vesicles

**Figure 4:**
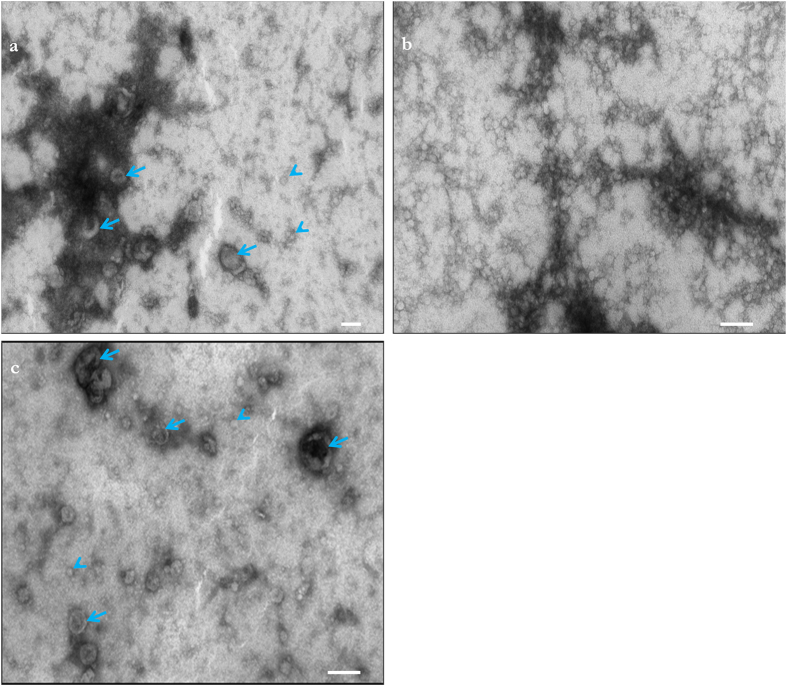
Transmission electron microscopic examination of heparin-purified EVs . All preparations were isolated from 1 ml of concentrated conditioned media from 293T cells. **(a)** Ultracentrifuged EVs. **(b)** Commercial kit-purified EVs. **(c)** Heparin-purified EVs. Scale bars = 100 nm. Arrows point to large EVs (~50-100 nm) and arrowheads point to small EVs (< ~ 50 nm). Note: It is unclear which of the structures observed with the commercial kit are EVs or if some are a component of the proprietary precipitating reagent.

To directly examine the morphology of heparin-purified EVs in comparison to other methods of EV isolation we performed transmission electron microscopy (TEM). EVs were obtained by UC, the commercial kit and direct purification from 293T cell conditioned media using heparin affinity and examined by TEM. Resuspended pellets from UC EVs showed the expected round-shaped vesicles with a size range of approximately 30-200 nm and some clumping of vesicles (Fig. 4a). On the other hand, the EM profile of vesicles isolated with the commercial kit was very uniform, containing small and round particles (~30-50 nm), which appeared to be connected to each other over a large network (Fig. 4b) and EV**-**looking structures were difficult to identify. EVs isolated using heparin affinity had a size distribution similar to that of the UC samples (Fig. 4c). Importantly, the heparin-purified EVs appeared to have typical size and morphology obtained with UC-purified EVs (Fig. 4c and Supplementary Fig. 9a-c).

### Heparin-purified extracellular vesicles are internalized by U87 cells

**Figure 5:**
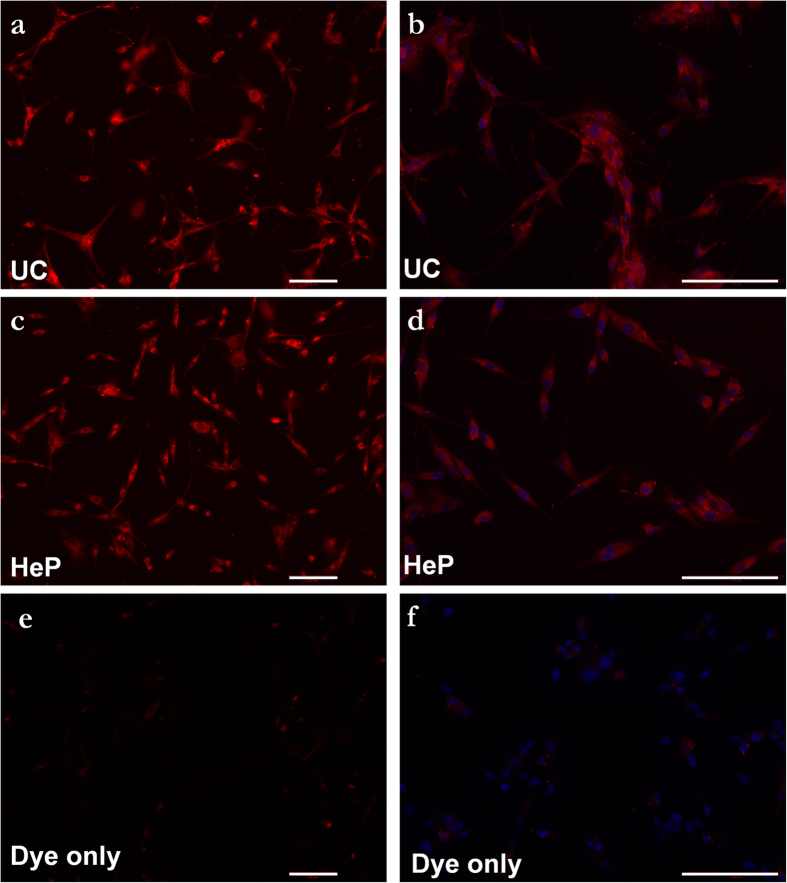
Heparin-purified EVs are internalized into cells. 293T-derived extracellular vesicles from **(a, b)** ultracentrifuged and heparin-purified **(c, d)** samples were labeled with red fluorescent lipid dye (see methods) and incubated with recipient U87 glioma cells to visualize internalization. After 60 minutes of incubation at 37 °C cells were fixed in formaldehyde, nuclei stained with Dapi and imaged using a fluorescence microscope. For control, a dye-only sample with no EVs was added to cells **(e, f)**. Scale bar = 110 μm.

EVs derived from 293T cells were heparin-purified or UC isolated. Next, EVs were membrane-labeled with a ceramide-conjugated red fluorescent dye. Unbound dye was removed using size exclusion centrifugal columns. Additionally, to control for incomplete dye removal, we column purified dye in the absence of EVs. Next labeled EVs from either purification method or the dye-only sample were added to wells of recipient U87 human glioma cells. We show that after 60 minutes of incubation with U87 cells, EVs isolated by either method entered U87 cells (Fig. 5a-d), suggesting that the heparin purification method retains EV functional integrity. In comparison, only minimal labeling was observed in the EV-free, dye-only sample (Fig 5e,f).

## Discussion

Purification and classification of extracellular membrane vesicles remains a challenge and here we show that heparin-coated beads can be used to purify EVs released from normal and cancer cells, and importantly from human blood plasma. We first analyzed the heparin-mediated isolation process of EVs released by 293T cells by NTA and found a satisfactory 60% recovery. We supported the NTA data (Fig. 1) in the following ways and additionally compared the heparin method directly to UC isolation (as well as other isolation methods): (1) we detected an established EV marker, Alix, on the isolated samples (Fig. 2a); (2) we determined the RNA yield and profile extracted from EVs (Fig. 2c); (3) we measured RNA transcript levels (Figs. 2d and 3b); (4) we analyzed morphology by transmission electron microscopy (Fig. 4); (5) we showed functional uptake of purified EVs in a cell culture assay (Fig. 5).

The purity of EV samples derived from cultured cells is very important for basic biological research which intends to assign functional activity to a given population of EVs. Co-precipitating contaminants such as protein, lipid or nucleic acid entities/aggregates could influence these biological activity assays, leading to uncertainty in data interpretation. When we probed nitrocellulose membranes containing EV proteins from the different isolation methods with an anti-BSA antibody, intense signal was observed in the UC samples (Supplementary Fig. 4), and BSA contamination in EV UC preparations has been previously reported^14^. BSA levels were consistently lower in heparin-purified samples as compared to UC. Importantly, the heparin purification process of EVs was amenable to biological assays, as we were able to show uptake of fluorescently labeled EVs into recipient cells (Fig. 5). TEM analysis of heparin-purified EVs displayed EVs with morphology typical of UC preparation, further supporting our isolation method (Fig. 4). Furthermore we also show that mRNA can be recovered from heparin purified EVs from human plasma, so this procedure may be applied to a variety of biofluids (Fig. 3). We switched our heparin affinity matrix from agarose beads (Figs. 1, 2) to the magnetic beads used in fig. 3 for the reason that our downstream goals with these samples were different and thus required different reagents. We used salt elution in our cell culture media experiments (Figs. 1, 2) in order to demonstrate that our method can result in functional EVs (Fig. 5), as cell-culture derived EVs are commonly utilized in basic research to gain knowledge of the physiological function of EVs. For plasma/biofluid EV isolation (Fig. 3), most researchers are not interested in extracting intact EVs, and they are focused on the biomarker components carried by the EV. With this in mind, we switched to magnetic beads which allowed us to directly extract RNA using Qiazol addition (agarose beads were not compatible with this reagent). This obviated the need for overnight high salt elution and reduced the protocol time by approximately 16 h. We also did not calculate % recovery of input using NTA as we did with cell culture media (Fig. 1), as the complexity in blood of various particles in the same range of EVs confounds accurate EV particle number. We did however directly compare the levels of two transcripts after isolation of EVs with UC or heparin affinity from the same volume of plasma (Fig. 3).

Another issue with co-precipitating proteins in EV preparations is that normalization to total protein amount may not correlate with EV numbers across different sample types. A recent report developed a protocol to measure both nanoparticle tracking analysis counts as well as protein amounts in EV samples. They found that sucrose-cushion purified samples had relatively high particle (EV) to protein ratios, while UC samples had much lower particle to protein ratios^14^. These results are important because they suggest that normalization to protein amounts may not be optimal to normalize for true EV numbers, as protein levels associated with EVs vary for different sample types (e.g. plasma vs. CSF) and preparation procedures. Similarly, a recent report showed that using size exclusion chromatography could effectively remove contaminating proteins, significantly reducing the protein concentration in the EV preparation^16^. Our data supports these findings, as when we normalized loading of heparin and UC samples to NTA counts, there was much higher protein content in the UC sample (Fig 2a,b ).

Two important parameters of any purification scheme are yield and scalability. We found that the yield of RNA using heparin purification was similar to that for UC (from the same input volume of media) as well as a commercial kit (Fig. 2c). Affinity purification is considered a more scalable method than centrifugation-based purification^37^.

We have previously shown that adding heparin to EVs blocks their uptake by recipient cells^22^ and recently HSPG has been identified as a receptor on the cell surface required for EV internalization^24^. Indeed we demonstrate that EVs bind to heparin beads and that this binding is substantially inhibited when the EVs are pre-incubated with free heparin (Supplementary Fig. 2a). We also show specificity of EV binding to heparin by first allowing EVs to bind to the beads and then using heparinase to digest the heparin and release EVs from the beads. Finally, we incubated EVs with the magnetic and agarose support matrix without conjugated heparin as a control for nonspecific binding (Fig. 3 and Supplementary Fig. 2b). We attempted to identify putative heparin binding ligands on the EV surface using peptide mass fingerprinting (PMF) as currently no receptor mediating the EV/HSPG interaction has been described. We chose to isolate the most intense bands (65 and 70 kDa) on Coomassie stained gels of heparin-purified EVs. Receptor proteins binding to heparin may be enriched on the isolated EVs, although we recognize that much of the proteins at this molecular weight may have been BSA (BSA was the major contaminant identified by mass spectrometry). We also were aware of studies showing heat shock proteins (HSPs) on the EV membrane and that some of these have heparin-binding domains^38^,^39^,^40^. Despite the BSA presence, peptide mass fingerprinting (PMF) predicted many other EV-associated proteins in this mass range. Our data suggests some interesting candidates (e.g. histones, heat shock proteins, and annexin which are known to be expressed on the EV surface^36^,^41^,^42^). Although we have demonstrated some receptor(s) which may contribute to HSPG binding of the 65 and 70 kDA bands, it will be of interest to analyze the other bands on the gel (Supplementary Fig. 6) to identify other potential heparin-binding receptors on the EV surface. It will also be important to verify these proteins on EVs by immunoblot analysis as well as functional blocking studies to investigate possible interactions with heparin/heparan sulfate proteoglycans. Finally, it should be noted that although many of the proteins with heparin-binding affinity identified as hits by mass spectrometry were found in the EV ExoCarta database, they may also be co-purification proteins, not necessarily directly associated with EVs.

Much of the biological activity as well as the biomarker potential of EVs is in their RNA content, e.g. mRNA, miRNA^43^,^44^. We showed by qRT-PCR that transcripts such as EGFR, GADPH and cMyc are detected using RNA extracted from EVs purified with heparin beads. Interestingly, of the transcripts examined from different EV isolation procedures, the values for heparin-purified samples was most similar to sucrose density gradient purified samples, which is provides a high level of vesicle purification. Compared to sucrose and heparin-purified samples, some of the transcripts had higher levels (e.g. cMyc, RPL11) for UC and the commercial kit, which is known to work by a precipitation method. Using these procedures it is possible that some of the transcripts may come from non-EV or disrupted EV sources. Alternatively, the heparin-based purification (and sucrose density gradient) may have isolated specific populations of EVs. We also isolated EVs from plasma samples using streptavidin coated magnetic beads bound to biotin-heparin. We detected mRNAs (GAPDH, RPL11) at similar levels as recovered by ultracentrifugation. Magnetic beads provide a fast and easy platform for isolation of EVs by heparin affinity, and this technology may be applicable to a variety of other biofluids, such as cerebrospinal fluid and urine, although further experimentation will be required to confirm this predication.

It is important to note that some proteins in media and biofluids can bind heparin. To reduce competition for binding sites on the heparin matrix we used 100 kDa molecular weight cutoff ultrafiltration devices before mixing samples with heparin beads. While this should remove many of the heparin binding proteins, in the future it may be useful to include a size exclusion chromatography pre-step (as reported^45^) to separate the megadalton EVs from smaller heparin binding proteins for further purification by heparin affinity. Importantly the method requires no expensive equipment or training with ultracentrifuges and provides EVs with biomarker and biological functional potential. Although in some instances (Fig. 2), we used ultracentrifugation (a common technique in our laboratory) with the heparin-agarose beads to pellet EVs in order to concentrate the sample (when the sample was split into aliquots for comparative analysis), we also show that we can use ultrafiltration to concentrate and desalt the preparation (Fig. 5). Furthermore, we demonstrated with the magnetic beads that no post-processing is required (Fig. 3). The EV-associated contents (e.g. protein or RNA), can be extracted directly off the beads.

Several studies have put forth different methods of EV isolation^46^, each with its own benefits and drawbacks. For example, ultracentrifugation and EV precipitating methods provide relatively high EV yields, although, as mentioned, high levels of co-pelleting non-EV biomolecules can compromise purity. For example, a recent study used peptides which precipitate EVs by binding to heat shock proteins associated with the vesicles^47^. Co- precipitating material appears to have obscured identifiable EVs in transmission electron micrographs unless the samples were treated with proteinase K prior to examination. Alternative methods such as density gradient ultracentrifugation, size exclusion chromatography, and affinity chromatography generally yield higher sample purity. Tauro *et al*. used Ep-Cam immunofinity to isolate exosomes from a transformed colon cancer cell line, LIM1863^10^. Using this approach, the authors performed extensive proteomics on the isolated EVs and showed that the process enriched for EV-specific proteins compared to UC. However, the authors only tested this on one cell line and only tested isolation in serum-free conditions. Chen *et al* used size exclusion high pressure liquid chromatography to isolate mesenchymal stem cell-derived EVs. The main similarities with our approach are the affinity purification of intact lipid structures with surface molecules that have an affinity for heparin (in our studies) and the fact that these methods can be scaled to increase the number of captured EVs. In contrast to these studies, we believe that this method will purify vesicles that are released from a variety of cell lines and not limited to the surface expression of one or two markers. We do not believe that any one method is “best” for every EV application. One must consider a variety of factors including speed of isolation, purity of vesicles ( for downstream application), and equipment and technical skill available to the user’s laboratory. It may also be useful to co-opt heparin purification with other methods to further “polish” the EV sample.

In conclusion, we show that heparin-based affinity chromatography can be used to efficiently purify an abundant population of extracellular vesicles. It may be a useful protocol for both studies of functional activity of EVs as well as biomarker isolation from biofluids.

## Methods

### Cell lines

The human glioblastoma cell line U87 and cell line 293T were obtained from American Type Culture Collection (Manassas, VA) and cultured in high glucose Dulbecco’s modified essential medium (DMEM; Life Technologies, Grand Island, NY) containing 10% fetal bovine serum (FBS; Sigma, St. Loius, MO) and Penicillin/Streptomycin (10 IU ml− 1 and 10 μg ml− 1, respectively; Cellgro, Manassas, VA). HUVEC were provided by Drs. Francis W. Luscinskas and Kay Case, Cell Core Facility, Brigham and Women’s Hospital supported by NIH P01 Hl36028. HUVECs were cultured in gelatin-coated flasks in endothelial basal medium (Lonza, Allendale, NJ) supplemented with human epidermal growth factor (hEGF), hydrocortisone, GA-1000 (Singlequots from Lonza).

### Extracellular vesicle isolation

293T cells were grown for 24 hrs in 15-cm culture plates (~20 million cells/plate) in a total of 20 ml DMEM prepared with 5% EV-depleted FBS^8^. FBS was made free of EVs by overnight (16 h) ultracentrifugation at 100,000 x g. Next the EV-depleted FBS was sterile filtered using a syringe fitted with a 0.22 μm Millex-GV PVDF filters (Millipore, Billerica, MA). For each experiment, 60 ml of conditioned media (from 3 plates) was used to isolate EVs. The media was first centrifuged at 300 x g for 10 minutes to remove any cells. The supernatant was then transferred to a clean tube and centrifuged again at 2,000 x g for 15 minutes to remove other debris. The supernatant was again transferred to a clean tube and filtered through a 0.8 μm filter (Millipore). At this point 60 ml of filtered media was concentrated down to 3 ml by centrifuging at 1000 x g for 10 minutes using a 100 kDa MWCO ultrafiltration device (Amicon® Ultra-15, Millipore). One ml of concentrated conditioned media each was used as input for all three isolation methods: heparin purification, ultracentrifugation, and ExoQuick-TC^™^ (referred to in the text as a commercial kit).

For heparin purification of EVs, one ml of Affi-Gel^®^ Heparin Gel (Bio-Rad, Hercules, CA) was washed twice with phosphate buffered saline (PBS), pH 7.2. On day one, 1 ml of concentrated conditioned media was added to the beads, inverted three times and incubated overnight on a tube rotator at +4 °C to allow binding of EVs to the heparin-coated beads. On day two, heparin beads were spun at 500 x g for 5 minutes and the supernatant (unbound fraction) was collected. Heparin-coated beads were washed three times with PBS and each wash supernatant was saved. Lastly, one ml of 2 M NaCl in PBS (final salt concentration 2.15 M) was added to the beads and incubated overnight at +4 °C on a tube rotator. On day three heparin-coated beads were centrifuged at 500 x g for 5 min. The supernatant, corresponding to the eluate, was collected and stored separately with the other supernatants at −80 °C for downstream analysis. The one ml of eluted sample was further processed using one of two different methods. In the first method, the EVs in the samples were pelleted by ultracentrifuging at 100,000 x g for 90 minutes and the pellet resuspended in PBS or the appropriate solution for the assay (e.g., RIPA buffer for SDS-PAGE or Qiazol for RNA extraction). In the second method, the 1 ml was concentrated and buffer exchanged to approximately 200 μl using 100 kDa MWCO Amicon^®^ Ultra-15 centrifugal devices. For some experiments where several aliquots were needed, we scaled up by using several heparin bead aliquots and concentrated the samples again using either ultracentrifugation or ultrafiltration.

Another ml of concentrated conditioned media was ultracentrifuged at 100,000 x g for 90 minutes and the pellet resuspended in PBS and used in parallel with heparin-purified EVs. The last ml of concentrated conditioned media was used to isolate EVs with the commercial kit, ExoQuick-TC™ (System Biosciences, Mountain View, CA). All three isolations were treated with DNAse I prior to nucleic acid extraction.

For heparin-based purification of EV using magnetic beads,. biotinylated heparin (Sigma-Aldrich, St. Louis, MO) was allowed to bind to MyOne Streptavidin CI Dynabeads (Invitrogen, Carlsbad, CA) during an incubation for 30 min at room temperature, at a concentration of 100 μg of heparin to 1mg of beads. Following this incubation, the heparin containing beads were separated from the unbound heparin using the DynaMag-2 magnet. The unbound heparin was removed from the beads using 100 uL of PBS, pH 7.4. 300 μl of heparin-magnetic beads were allowed to bind with 500 μl of pre-washed plasma samples and were incubated at 4 °C overnight. The unbound was removed using the magnet and the beads were washed three times with PBS. Bound EVs were lysed directly on the beads (no elution or further processing as with heparin agarose beads) using 700 μl of Qiazol and it was preceded to the RNA extraction as described in *Extracellular vesicle nucleic acid extraction*. GAPDH and RPL11 mRNAs were detected in heparin-bead isolated as well as UC samples.

In some experiments, sucrose gradient purification of EVs was performed on one ml of concentrated media. The sucrose density gradient purified EVs were obtained by overlaying an 8%/ 30%/ 45%/ 60% sucrose step gradient with concentrated conditioned media from 293T cells. The gradient was ultracentrifuged in a MLS-50 swinging bucket rotor for 38 min at 50,000 rpm with brake setting at 8 (almost coasting). Fractions 3-7 were collected and diluted with PBS to a total volume of 2.3 ml and place in ultraclear tubes for MLA-55 rotor. The EVs were pelleted at 100,000 x g for 1 h and the pellet resuspended in PBS and stored at −80 °C.

### Extracellular vesicle nucleic acid extraction

Extracellular vesicles from 1 ml of concentrated conditioned media were purified by heparin beads, eluted in 1 ml of 2 M NaCl in PBS, diluted to 2.3 ml with PBS 1x and then ultracentrifuged at 100,000 x g for 90 min and the EV pellet lysed in 700 μl of Qiazol. RNA was extracted using the miRNeasy kit which reliably isolates mRNAs and miRNAs but, if warranted, includes further steps to enrich for miRNA (Qiagen, Valencia, CA) according to the manufacturers’ recommendations for total mRNA isolation. Quantity and size range of the nucleic acid were evaluated using the 2100 Bioanalyzer (Agilent, Santa Clara, CA) using the RNA 6000 pico chip which measures RNA species from 25 nucleotides to 6000 nucleotides We also isolated RNA from ExoQuick TC™ kit precipitated EVs, UC prepared EVs, and sucrose gradient-purified EVs and performed the same analysis as for heparin-purified EVs.

### SDS-PAGE gels, coomassie staining, immunoblot for Alix

One ml of concentrated media was either ultracentrifuged (UC), purified with the commercial kit (kit), purified with heparin coated beads (HeP), or by sucrose gradient (Suc). UC samples were resuspended in 100 μl of PBS and split into 3 aliquots for WB, total protein and total RNA analysis. Heparin purified and sucrose gradient-purified EVs were ultracentrifuged at 100,000 x g for 90 minutes in a MLA-55 fixed angle rotor and then the EV pellet was resuspended in 100 μl of PBS, RIPA or Qiazol and used for downstream analysis. Samples were loaded with equal amounts of total nucleic acid content as determined by the bioanalyzer, or EV number as determined by the NanoSight, and loaded on two separate SDS-PAGE gels in loading dye. One gel was used for Coomassie staining of total protein and the second gel was used to detect the exosomal marker Alix^2^. Coomassie staining was performed using GelCode Blue Staining Reagent (ThermoFisher, Waltham, MA) according to the manufacturer’s recommendations. In some cases, SDS-PAGE gels were stained using PlusOne™ silver staining kit (GE Healthcare) according to the manufacturer’s instructions. To determine EV purity we used a nanoparticle to total protein ratio calculation. First, we determined the amount of total protein in each lane using the “Gels” densitometry function of ImageJ. Each peak corresponding to a band on the gel was quantitated as area units using the wand (tracing) tool. The nanoparticle input per lane (2.1 × 10^10^) was divided by the sum of all peak values for each lane to get the nanoparticle:total protein ratio.

The immunoblot was performed using an Alix primary antibody (1:100; Santa Cruz, Santa Cruz, CA) with overnight incubation at +4 °C and a horse radish peroxidase (HRP)-conjugated secondary antibody (1:5,000; GE Healthcare, Piscataway, NJ). The membrane was washed three times with PBS-Tween and visualized using chemiluminescence detection of HRP activity with a Pierce Supersignal Western Pico Chemiluminescent Substrate kit (Pierce, Rockford, IL) followed by exposure of the membrane to autoradiography film (Denville Scientific, Metuchen, NJ).

### qRT-PCR

Total RNA was eluted in 30 μl RNAse-free water and 14 μl was used as input for cDNA reaction using the SuperScript® VILO™ (Invitrogen, Carlsbad, CA) in a total of 20 μl. One μl of the cDNA was used for each qRT-PCR reaction. TaqMan primers and probes from Applied Biosystems were used to detect human GAPDH, EGFR, LINE1, RPL11, CD63 and cMyc RNA. All qRT-PCR reactions were performed in 25 μl reactions using the fast TaqMan MasterMix (Applied Biosystems, Foster City, CA). Amplifications conditions consisted of 50 °C, 2 min; 95 °C, 10 min; 40 cycles of 95 °C, 15 s, 60 °C, 1 min on standard mode and were performed using ABI PRISM 7500 (Applied Biosystems). Primer sequences from Applied Biosystems were as follows: EGFR - Hs01076078_m1 (spans exons); GAPDH-Hs03929097_g1 (within one exon); CD63 - Hs01041237_g1 (spans exons); RPL11-Hs00831112_s1 (spans exons); cMyc Forward CAACCCTTGCCGCATCCAC; cMyc Reverse AGTCGCGTCCTTGCTCGG; Fam labeled probe: AGCAGCGGGCGGGCACTTTGC ACT (spans exons); LINE1 Forward ACCCTACAAGCCAGAAGAGAGT; Reverse GGCTGGATATGAAA TTCTGGGTTGA; Probe FAMTCTTTAAGAATGTTGAATATTGGC (within one exon).

### Nanoparticle Tracking Analysis (NTA)

EVs were purified by heparin-coated beads, ultracentrifugation, or ExoQuick and quantified using the Nanosight LM10 (Malvern, Framingham, MA). Samples were diluted in PBS 1x from 1 ml of eluate, 100 μl of sedimented vesicles or 100 μl ExoQuick precipitated material, respectively. Each samples was recorded three times for 30 seconds and analyzed in Auto Mode using the 2.2 NTA software (Malvern).

### Heparinase treatment of beads

One ml of concentrated conditioned media was incubated with 1 ml of heparin-coated beads and incubated over night at +4 °C on a tube rotator. The day after beads were pelleted 500 x g for 5 min) and the supernatant was removed. Beads were then washed three times with PBS 1x and either treated with *Bacteroides Heparinase I* (New England Biolabs, Ipswich, MA; 60U/ml heparin beads) according to the manufacturer’s recommendations or mock treated (incubation buffer devoid of heparinase) for 1 hr at 30 °C. Beads were pelleted and the supernatant was used to count the number of eluted EVs using NTA.

### Control bead binding assay

The Affi-Gel® Heparin Gel is based on a cross-linked agarose gel support. To control for non-specific binding of EVs to this support, we used Affi-Gel 10 (Bio-Rad). This cross-linked agarose gel contains a spacer with an activated N-hydroxy-succinimide (NHS) ester to covalently link ligands to the gel via an amide bond. We first inactivated the NHS ester by incubating beads in 100 mM Tris buffer pH 7.4 for 1 h so as to prevent coupling of EV transmembrane proteins to the bead surface. Next incubations of 293T-derived EVs with these control beads as well as the Affi-Gel® Heparin Gel beads (also incubated with Tris buffer as above) were performed and all fractions and elutions were collected and analyzed by NTA.

### Quantitation of EVs with high and low affinity for heparin

293T-derived EVs were incubated with Affi-Gel® Heparin Gel as described above in **Extracellular vesicle isolation.** Both the unbound and eluted (eluted with 2M NaCl in PBS, final 2.15M NaCl) fractions were collected. Salt was removed from the eluted sample and exchanged with PBS using a 100 kDa MWCO ultrafiltration device (Millipore). Next, separate aliquots of fresh Affi-Gel® Heparin Gel was mixed with the eluted fractions and unbound fractions from round 1. A second round of incubation, washes and elution was performed and particle counts performed by NTA.

### Functional uptake assay of heparin-purified extracellular vesicles

Heparin bead-eluted EVs from 293T cells were concentrated and exchanged with PBS using 100 kDa MWCO ultrafiltration device (Amicon® Ultra-15, Millipore). For comparison, UC prepared EVs from 293T cells were also resuspended in 100 ul of PBS. Next, EVs were labeled with BODIPY® TR Ceramide (Life Technologies) according to the manufacturer’s instructions. Briefly, 1 μl of 1 mM BODIPY® TR Ceramide was added to 100 ul of EV sample in PBS. To control for background staining in the uptake assay, 1 μl of 1 mM BODIPY® TR Ceramide was added to 100 ul of PBS without EVs. Next samples were incubated at 37 °C for 20 min. Excess unincorporated dye was removed using Exosome Spin Columns (MWCO 3,000, Life Technologies) according to manufacturer instructions. Next labeled EVs (in about 90 μl of PBS) or the dye-only sample were then added to U87 cells in glass chamber slides, (20,000/well) and incubated at 37 °C for 60 minutes. Unbound EVs were washed away once with PBS. Cells were fixed in 4% PFA in PBS for 10 minutes at room temperature. Fixed cells were washed again in PBS, DAPI stained and coverslipped. Images were acquired using a fluorescence microscope using the following setup: Dapi detection was done using a filter with excitation spanning 340-380 nm, a dichroic mirror at 400 nm, and emission filter spanning 435-485 nm. For BODIPY we used an excitation filter with 530-560 nm wavelength detection, a dichroic mirror at 570 nm, and emission filter spanning 590-650 nm.

### Electron microscopy

Five microliters of each EV sample (heparin affinity purified samples were further concentrated with a 100,000 x g UC step) was adsorbed for 1 minute to a carbon coated grid that had been made hydrophilic by a 30 second exposure to a glow discharge. Excess liquid was removed with filterpaper (Whatman) and the samplesx were stained with 0.75% uranyl formate for 30 seconds. After removing the excess uranyl formate with filter paper the grids were examined in a JEOL 1200EX Transmission electron microscope or a TecnaiG^2^ Spirit BioTWIN and images were recorded with an AMT 2k CCD camera.

### Mass spectrometry analysis of proteins in HeP purified sample

The 65 and 70 kDa bands from a Coomassie stained gel of heparin purified EV sample from 293T culture media (Supplementary Fig. 6) were excised, placed in deionized sterile water, and submitted to the Harvard Medical School’s Taplin Biological Mass Spectrometry facility. Bands were analyzed using Orbitrap mass spectrometers (Thermo Scientific). Protein identification was predicted using the database search algorithm, SEQUEST.

### Statistical analysis

Statistical analyses were performed using Students *t*-test in Graph Pad Prism software.

## Author Contributions

C.A.M. and J.S. conceived of the study. C.A.M., L.B., B.A.T., J.S. and X.O.B. designed experiments and analyzed datas. L.B., C.A.M., W.C. and D.M. performed the experiments. N.A.A. helped with WBs. L.B. wrote the manuscript with input from all authors.

## Additional Information

**How to cite this article**: Balaj, L. *et al.* Heparin affinity purification of extracellular vesicles. *Sci. Rep.* **5**, 10266; doi: 10.1038/srep10266 (2015).

## Supplementary Material

Supporting Information

## Figures and Tables

**Table 1:** Top hits for 70 kDa band, Mass spectrometry results.

**Protein**	**Number of unique peptides matched**	**% Coverage**	**MW (kDa)**	**Membrane associated?**	In ExoCarta database?*	**Heparin binding domain?**
Annexin A1	14	50	38#	Yes	Yes	Yes^31^
Mucin-5B	12	2.5	596	Yes	No$	UK
Zymogen granule protein 16 homolog B	10	52	23	Yes	Yes	Yes^32^
Ig alpha-1 chain C region	7	19	38	Yes	Yes	UK
Tropomyosin alpha-1 chain	7	13.7	33	No	Yes	NA
Heat shock 70 kDa protein 1-like	7	14	70	Yes	Yes	Yes^35^
Heat shock 70 kDa protein 1A/1B	7	12	70	Yes	No$	Yes^35^
Actin, aortic smooth muscle	7	20	42	No	Yes	NA
Lysozyme C	6	20	16.5	No	Yes	NA
Cornulin	6	24	53	Yes	Yes	
Tropomyosin beta chain	6	22	33	No	No$	NA
Pyruvate kinase isozymes M1/M2	5	15	58	No	Yes	NA
Histone H4	4	42	11	No	Yes	NA
Prolactin-inducible protein	4	26	17	No	No	NA
Actin,						
cytoplasmic 1	4	18	42	No	Yes	NA
Troponin T, fast skeletal muscle	4	18	32	No	No	NA
Myosin-9	4	3	226	Yes	Yes	UK
Alpha-2-macroglobulin	4	2	163	No	Yes	NA

NA, not applicable UK, unknown.

^*^EV associated determined by publications displayed in ExoCarta.

^$^Exact match not found in database but other family members of the protein are in database.

^#^known to form dimers^3^.

**Table 2:** Top hits for 65 kDa band, Mass spec results.

**Protein**	**Number of unique peptides matched**	**% Coverage**	**MW (kDa)**	**Membrane associated?**	**EV associated?**	**Receptors w/ Heparin binding domain?**
T-complex protein 1 subunit beta	24	38	57	No	No$	NA
T-complex protein 1 subunit theta	24	38	60	No	Yes	NA
Pyruvate kinase isozymes M1/M2	17	34	58	No	Yes	NA
T-complex protein 1 subunit eta	16	34	59	No	No$	NA
T-complex protein 1 subunit delta	15	37	58	No	Yes	NA
Tubulin beta-2A chain	12	23	50	No	Yes	NA
Copine-8	11	27	63	Yes	Yes	NA
T-complex protein 1 subunit alpha	11	24	60	No	Yes	NA
Tubulin alpha-1A chain	10	36	50	No	Yes	NA
T-complex protein 1 subunit zeta	10	24	58	No	Yes	NA
RuvB-like 1	8	27	50	Yes	Yes	UK
D-3-phosphoglycerate dehydrogenase	8	19	57	No	Yes	NA
Aspartate--tRNA ligase, cytoplasmic	8	20	57	No	Yes	NA
Annexin A11	8	16	54	No	Yes	NA
EGF-like repeat and discoidin I-like domain-containing protein 3	8	18	54	No	Yes	NA
Centrosomal protein of 55 kDa	7	20	55	No	No	NA
Basigin	7	22	42	Yes	Yes	UK

NA, not applicable.

^$^Exact match not found in database but other family members of the protein are in database.
