# Supplementary material for: Heparin affinity purification of extracellular vesicles
Source: Sci Rep. 2015 May 19;5:10266. doi: 10.1038/srep10266 (PMC4437317; doi:10.1038/srep10266)
Supplement: Supporting Information [file srep10266-s1.pdf]

Supplementary Figures and legend

Heparin affinity purification of extracellular vesicles.

Leonora Balaj[1], Nadia A. Atai[1,2], Weilin Chen[1], Dakai Mu[1],
Bakhos A. Tannous[1], Xandra O. Breakefield[1], Johan Skog[3], Casey A. Maguire[1]

a

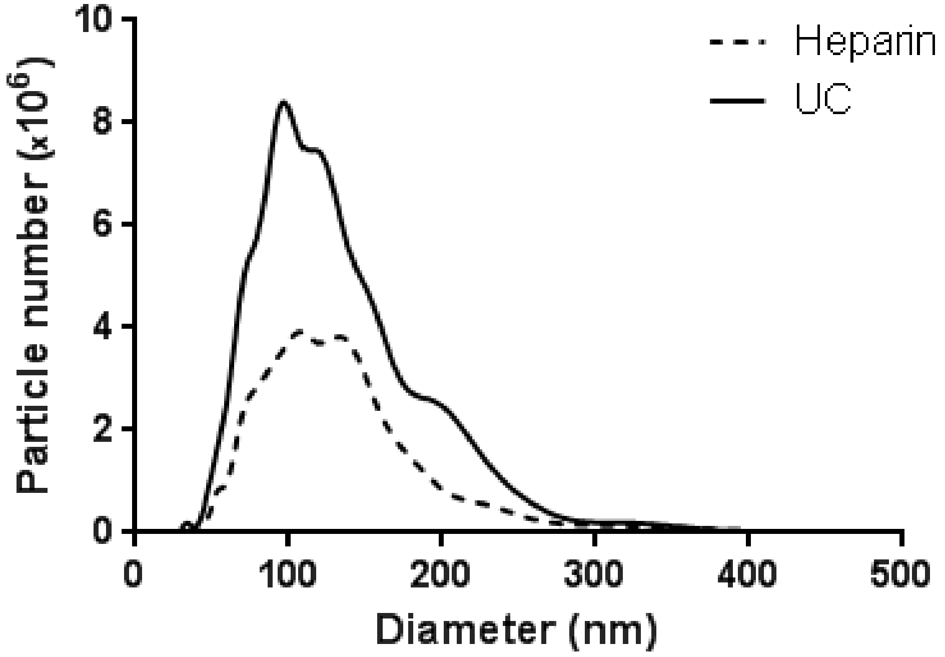

b

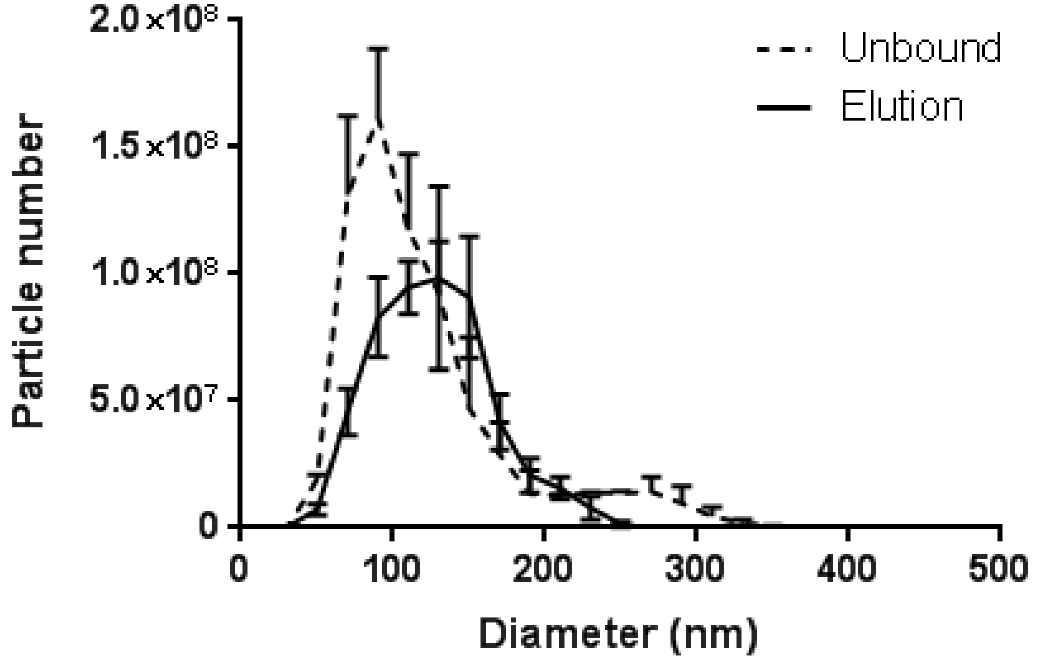

Supplementary Figure 1. Size distribution profiles of EVs. (**a**) EVs from 6 different biological experiments are plotted together and the average profile is shown. (**b**) NTA profiles of unbound and eluted EV fractions isolated using agarose heparin beads (n=3).

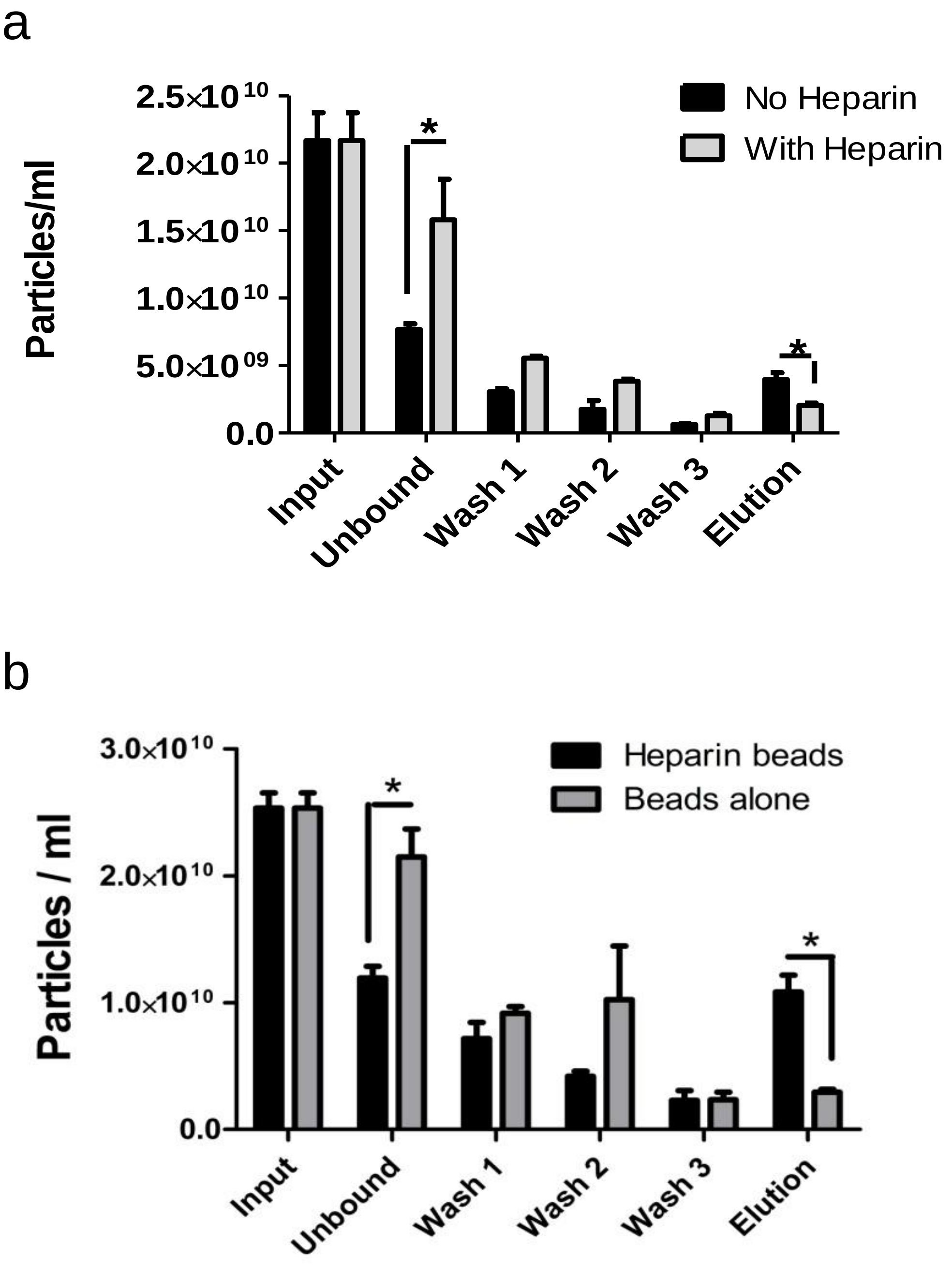

Supplementary Figure 2. EV binding to heparin-beads is specific. (**a**) EV were mock treated or incubated with 0.1 mg/ml soluble heparin for 1 hr at room temperature and then incubated with heparin beads overnight at 4°C. NaCl-eluted samples were analyzed using the NTA (**b**) 293T-derived EVs were incubated with either control agarose beads alone, or heparin beads and subjected to purification and NTA counts.

a

b

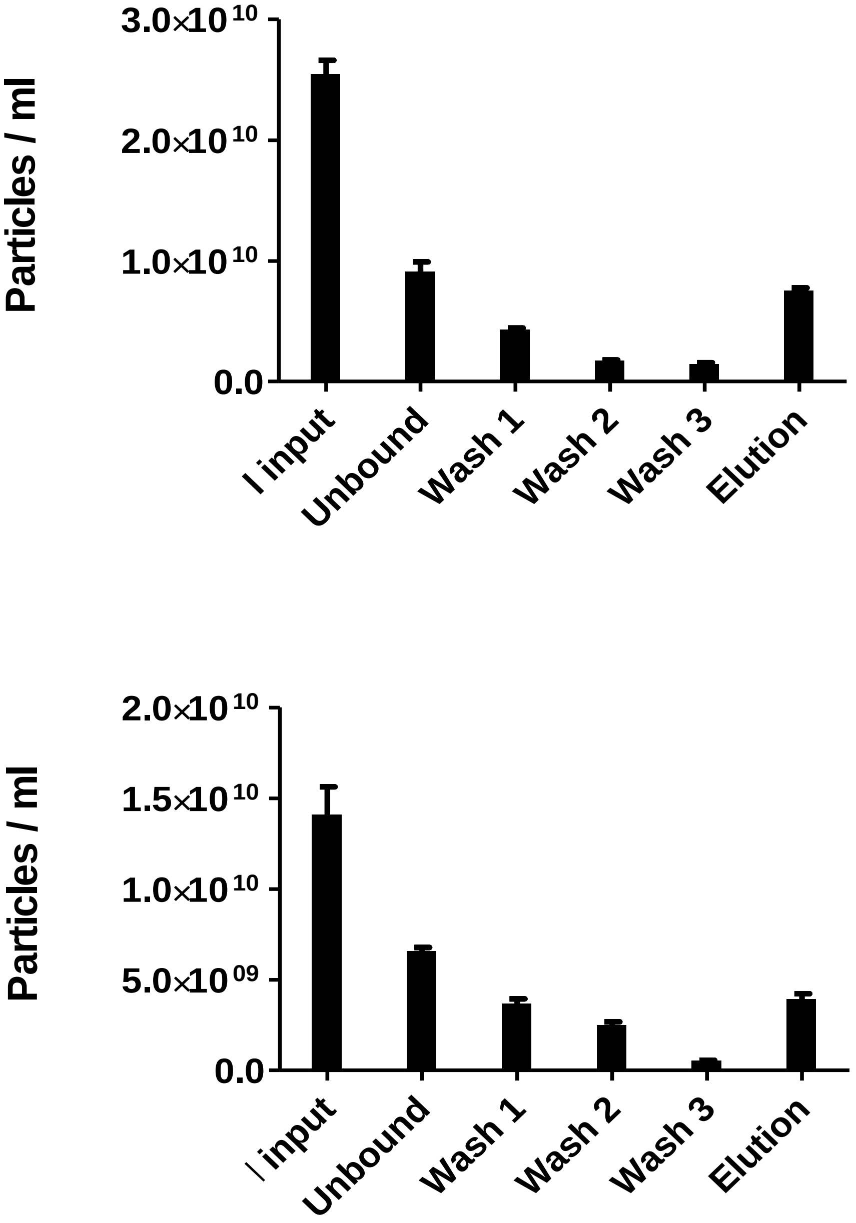

Supplementary Figure 3. Purification of EVs from other cell types using heparin beads.(**a**) Human glioblastoma cell line U87 and (**b**) human umbilical vein endothelial cells (HUVECs)-derived EVs were incubated with heparin beads, washed, and salt eluted. EVs recovery was assessed as above. The data is presented as mean ±s.d. (n=3).

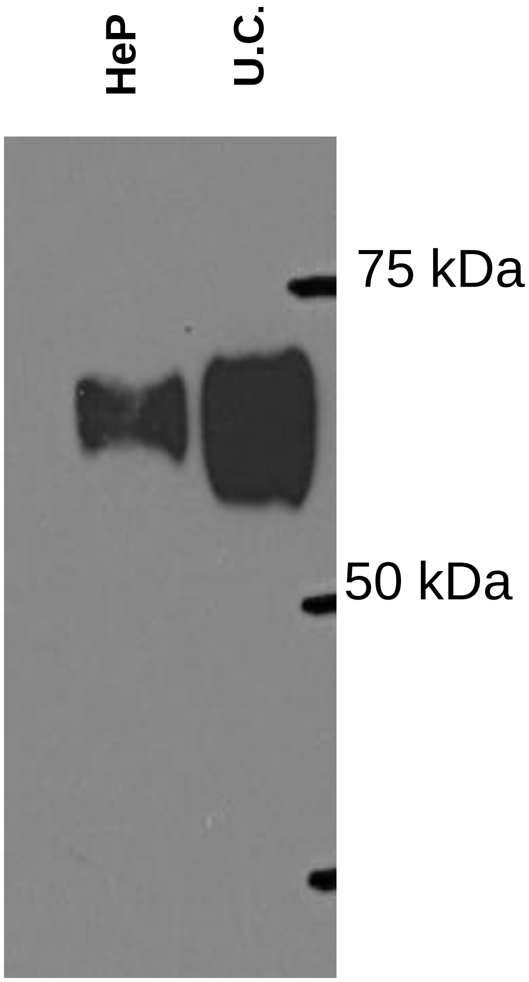

Supplementary Figure 4. Detection of BSA in EV samples. UC or heparin-purified samples were normalized for vesicle counts by NTA and subjected to SDS-PAGE and immunoblotting with an anti-BSA antibody.

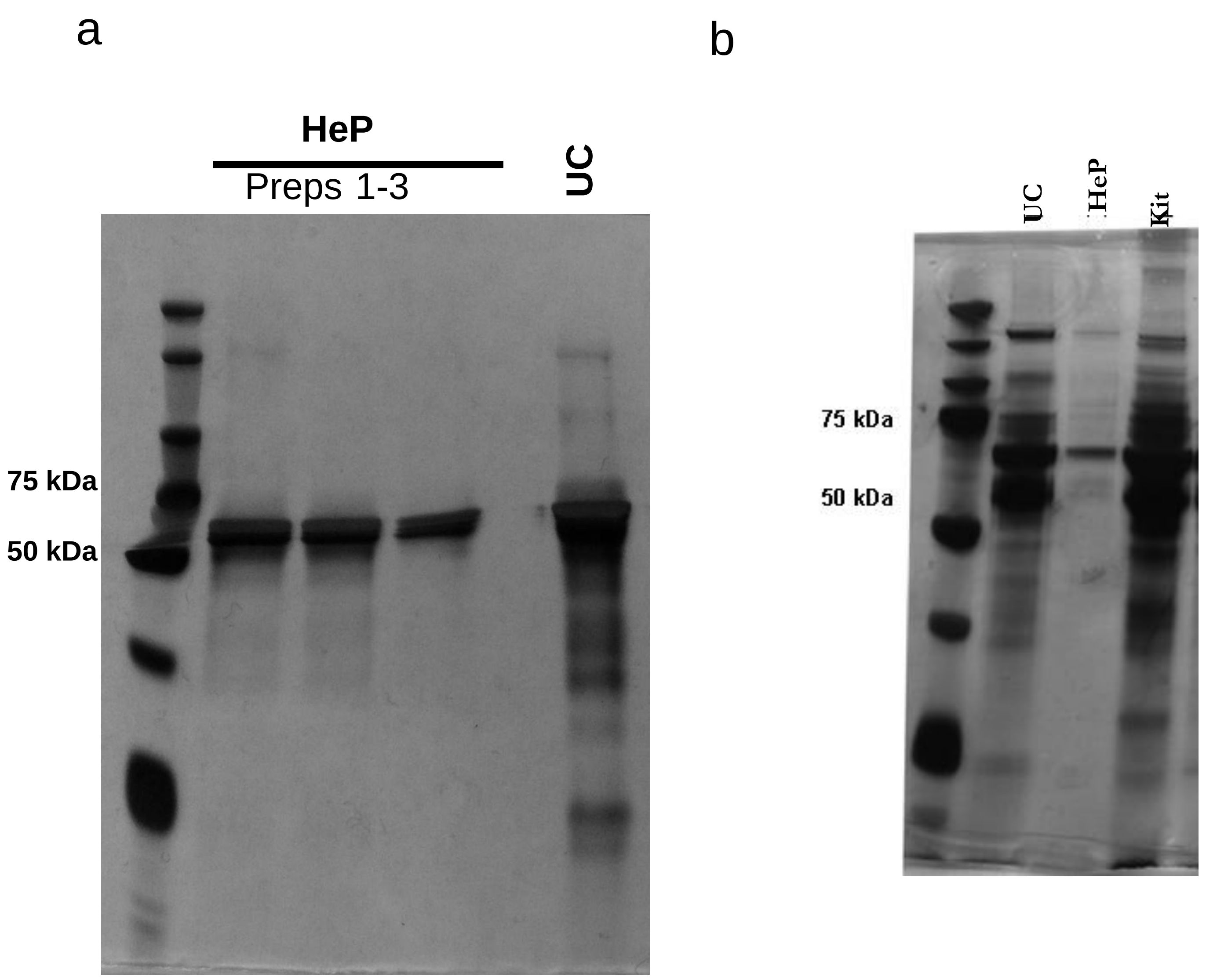

Supplementary Figure 5. Characterization of total proteins in EV preparations. (a) Silver stain analysis of electrophoresed EV samples obtained by heparin purification or ultracentrifugation. The loading was normalized to equal EV counts based on nanoparticle tracking analysis. **(b)** Recovered 293T-derived EVs purified with each of these methods had nucleic acid extracted and quantitated using a Bioanalyzer. The nucleic acid quantitation was used as loading normalization for SDS PAGE, in which 16 ng of nucleic acid was loaded per lane. Silver staining for total protein in each samples preparation.

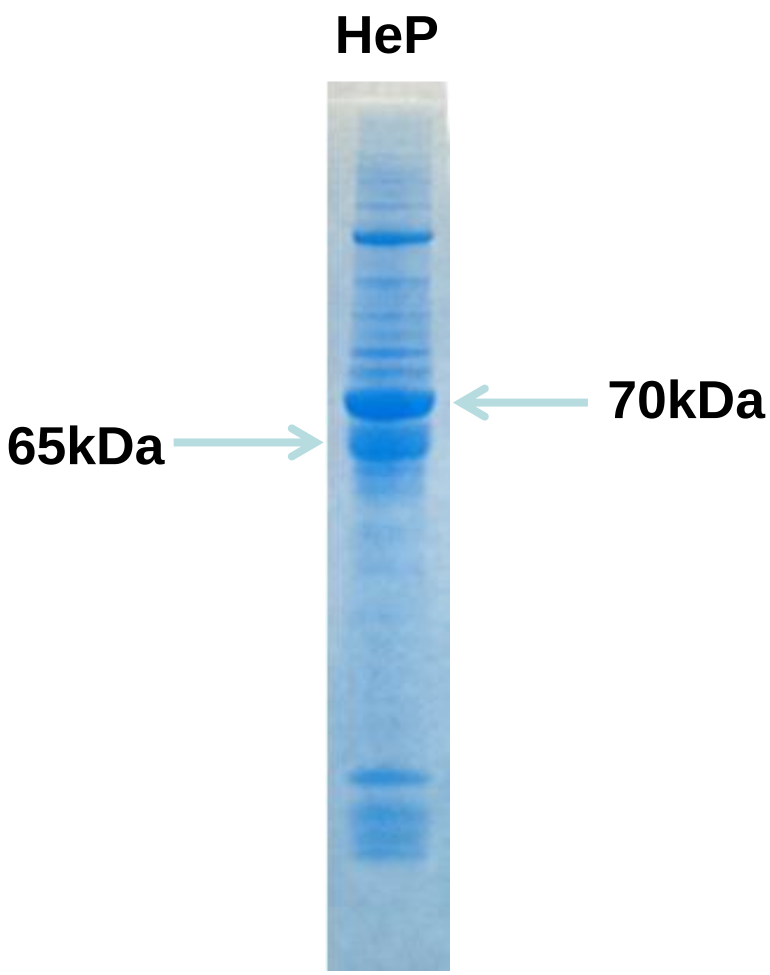

Supplementary Figure 6. Protein profiling of 293T EVs. EVs were isolated from culture media using heparin affinity and lysed in RIPA buffer to release proteins. Total proteins are loaded based on vesicle counts and analyzed by Coommassie Blue staining .The indicated bands were cut from the gel and analyzed with mass spectrometry.

HSP 70 immunoblot on human plasma derived EV samples for reviewers

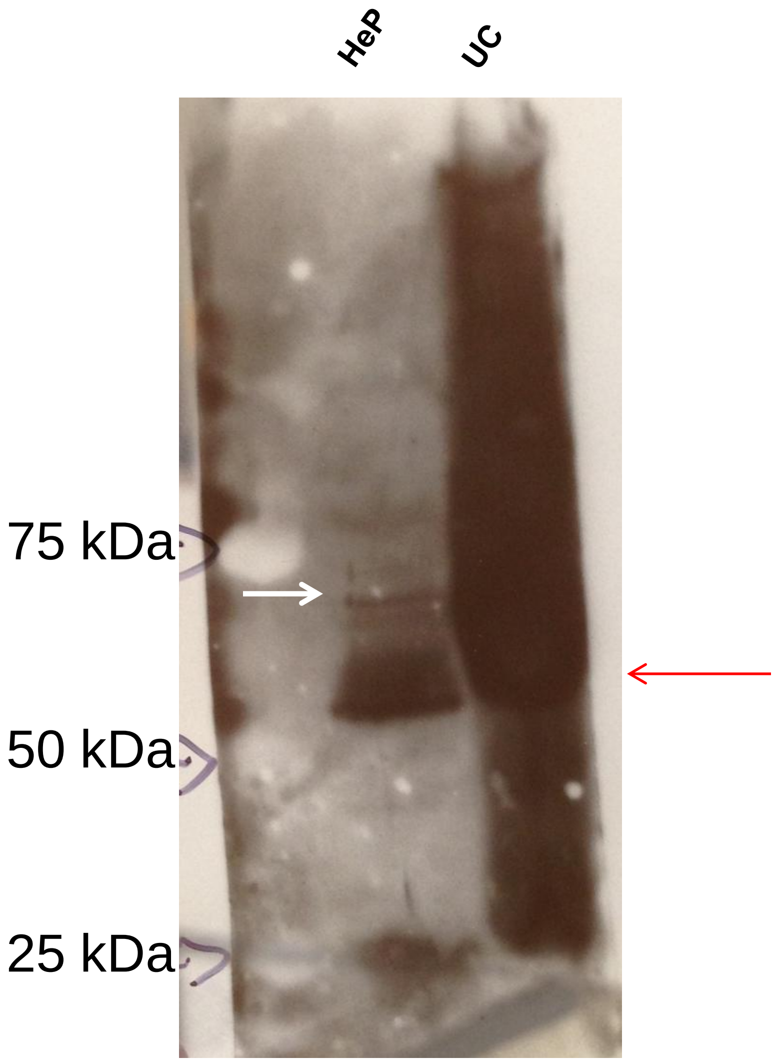

Supplementary Figure 7. Human plasma was purified according to Figure 3. Same samples as in Coomassie Gels. Immunoblot for HSP70 was performed. Specific band of 70kDa indicated by white arrow (seen only in HeP sample). Non-specific band (presumably albumin), red arrow (seen in both samples).

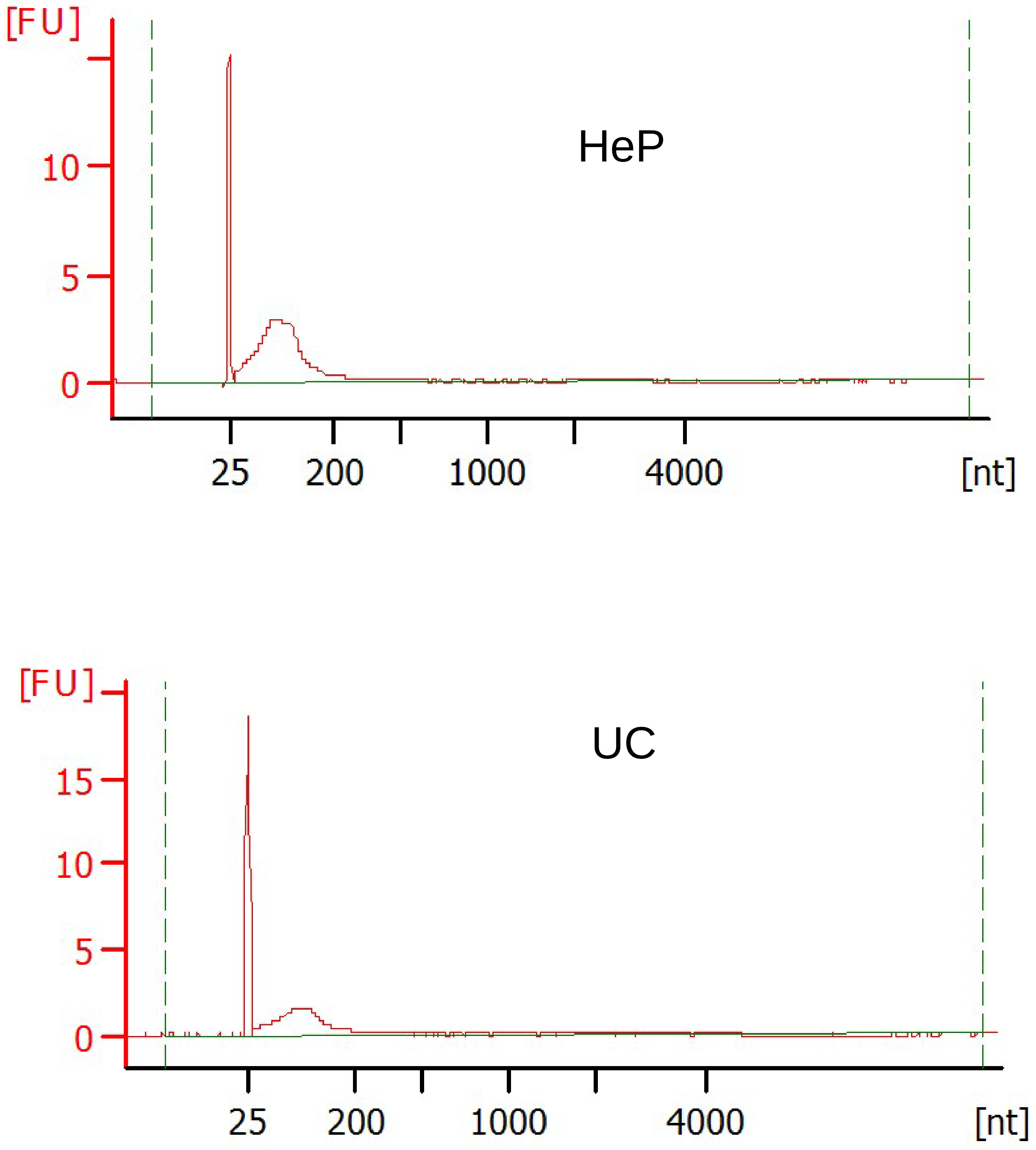

Supplementary Figure 8. Bioanalyzer profiles of the RNA from plasma samples. Two ml of plasma was washed with PBS using a nanofilter device and split into 3 aliquots and incubated overnight at +4C with biotin-heparin coated streptavidin beads. One aliquot was lyzed in Qiazol and used for RNA extraction. The data is generated using an RNA 6000 pico chip and indicates nucleotides (x-axis) and fluorescent units (y-axis).

Supplementary Figure 9a. Ultracentrifuged EVs

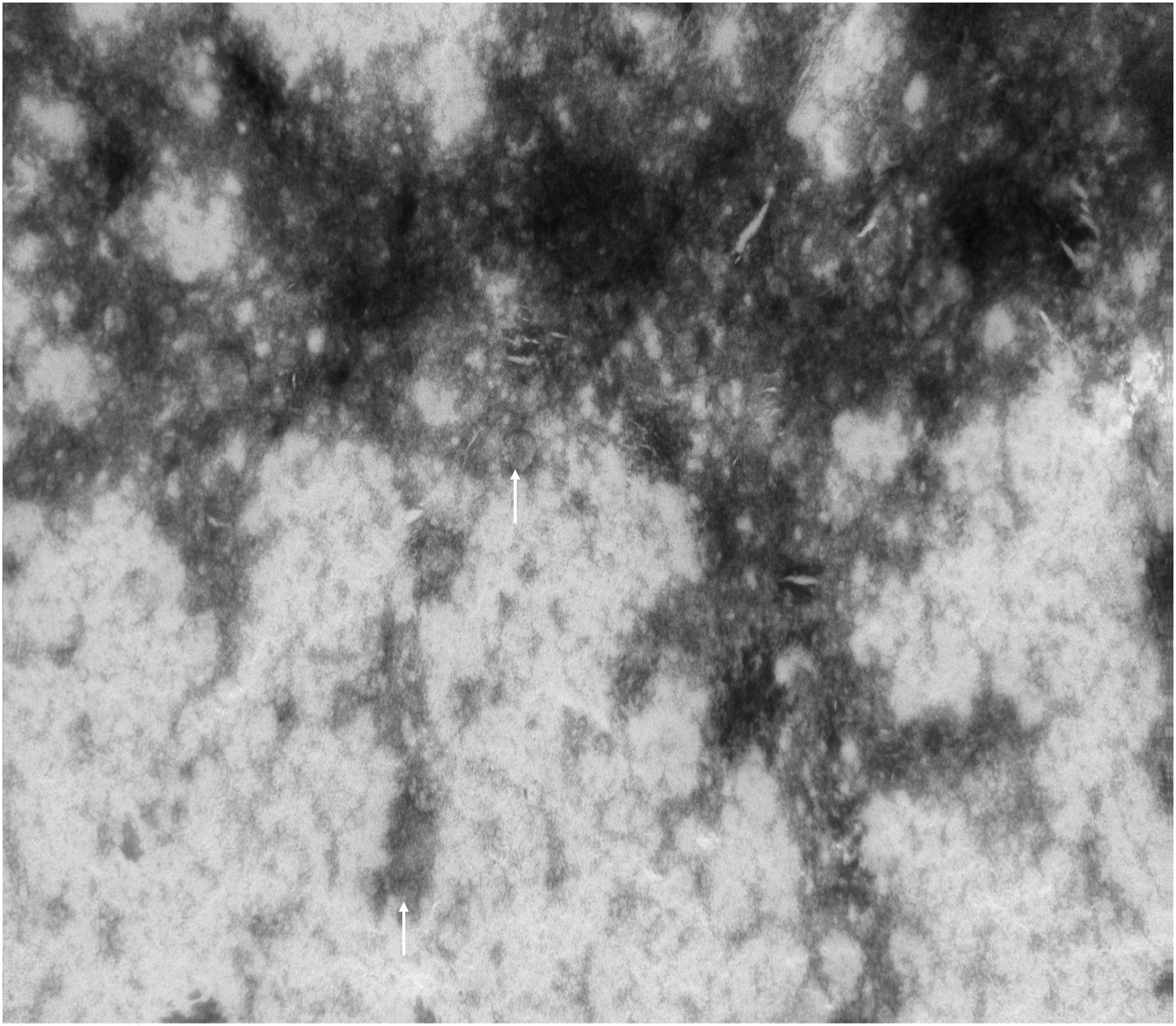

July 20 2012_002.tif
sample 1
Print Mag: 15100x @ 51 mm
15:57 07/20/12

100 nm
HV=80.0kV
Direct Mag: 20000x
AMT Camera System

Supplementary Figure 9a. Ultracentrifuged EVs

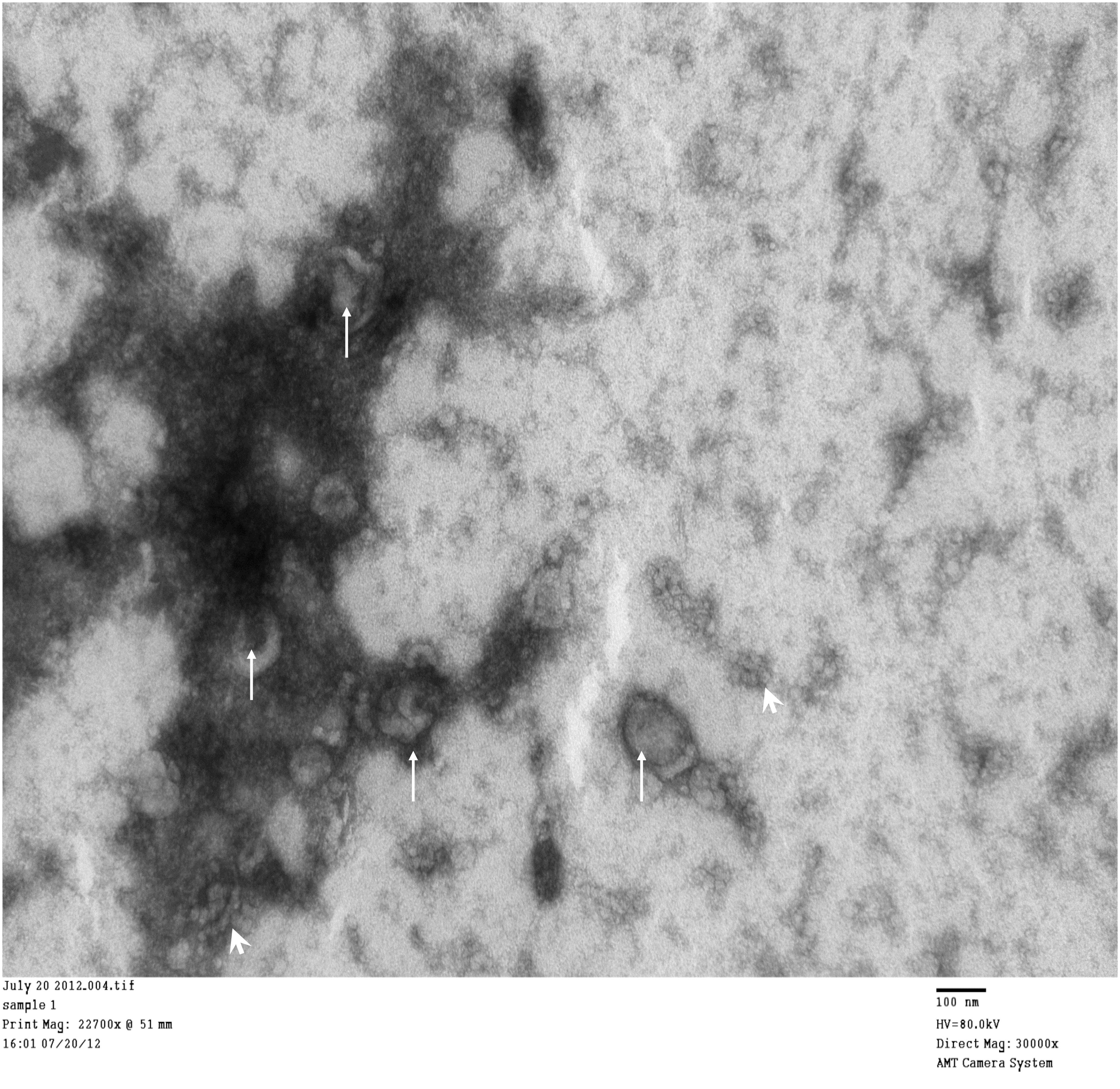

Supplementary Figure 9b. Kit EVs

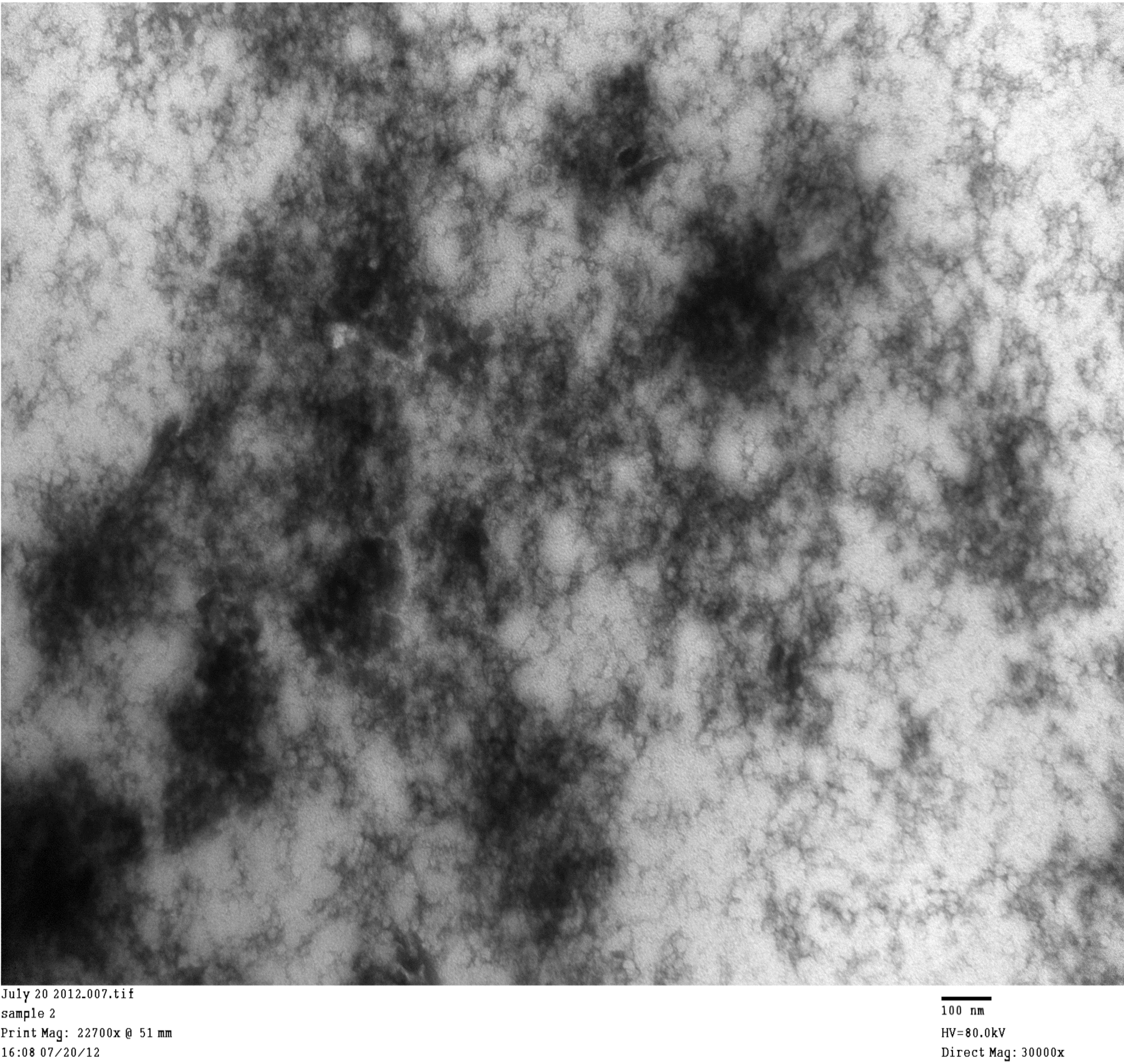

Supplementary Figure 9b. Kit EVs

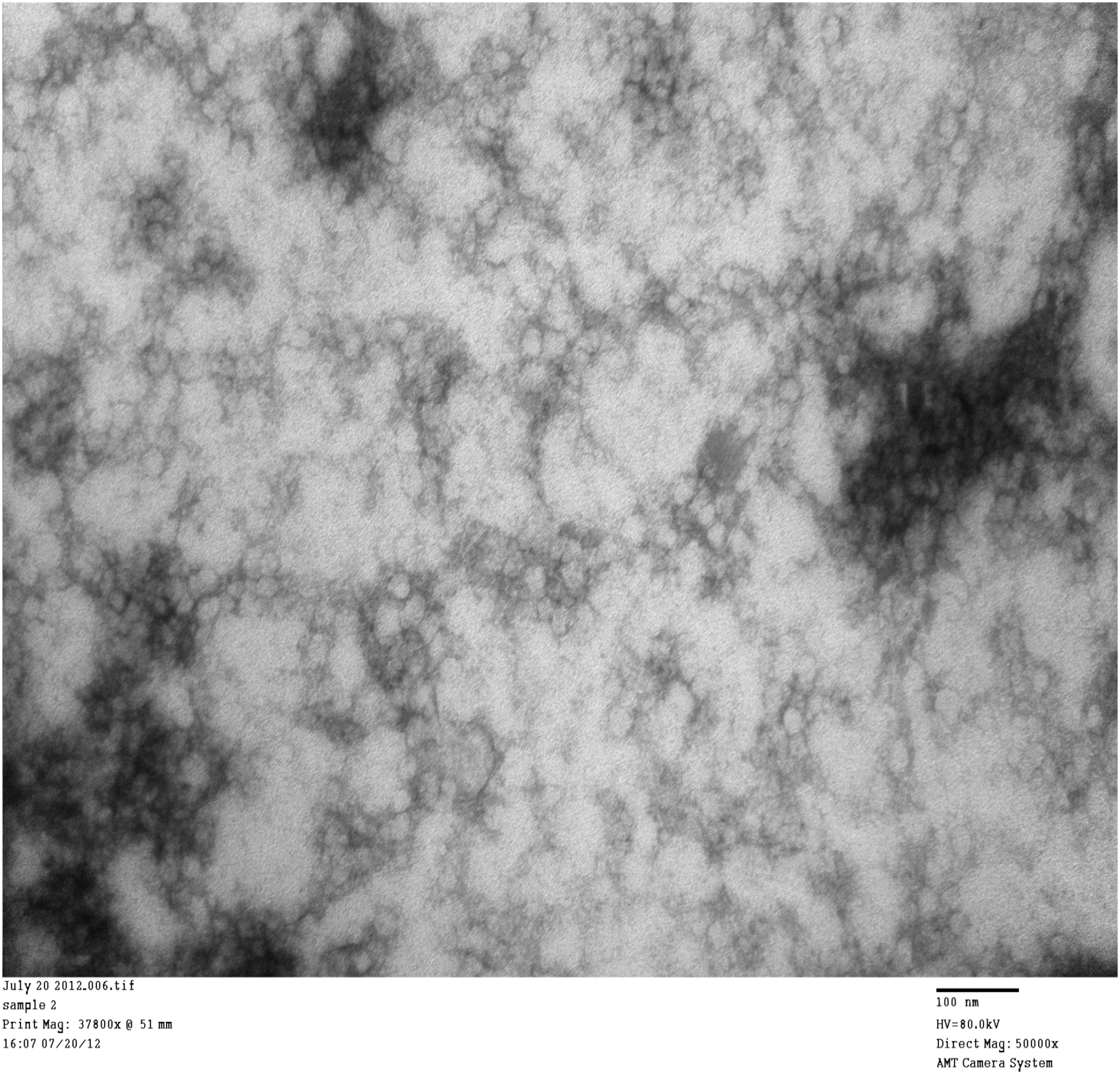

Supplementary Figure 9c. Heparin-purified EVs

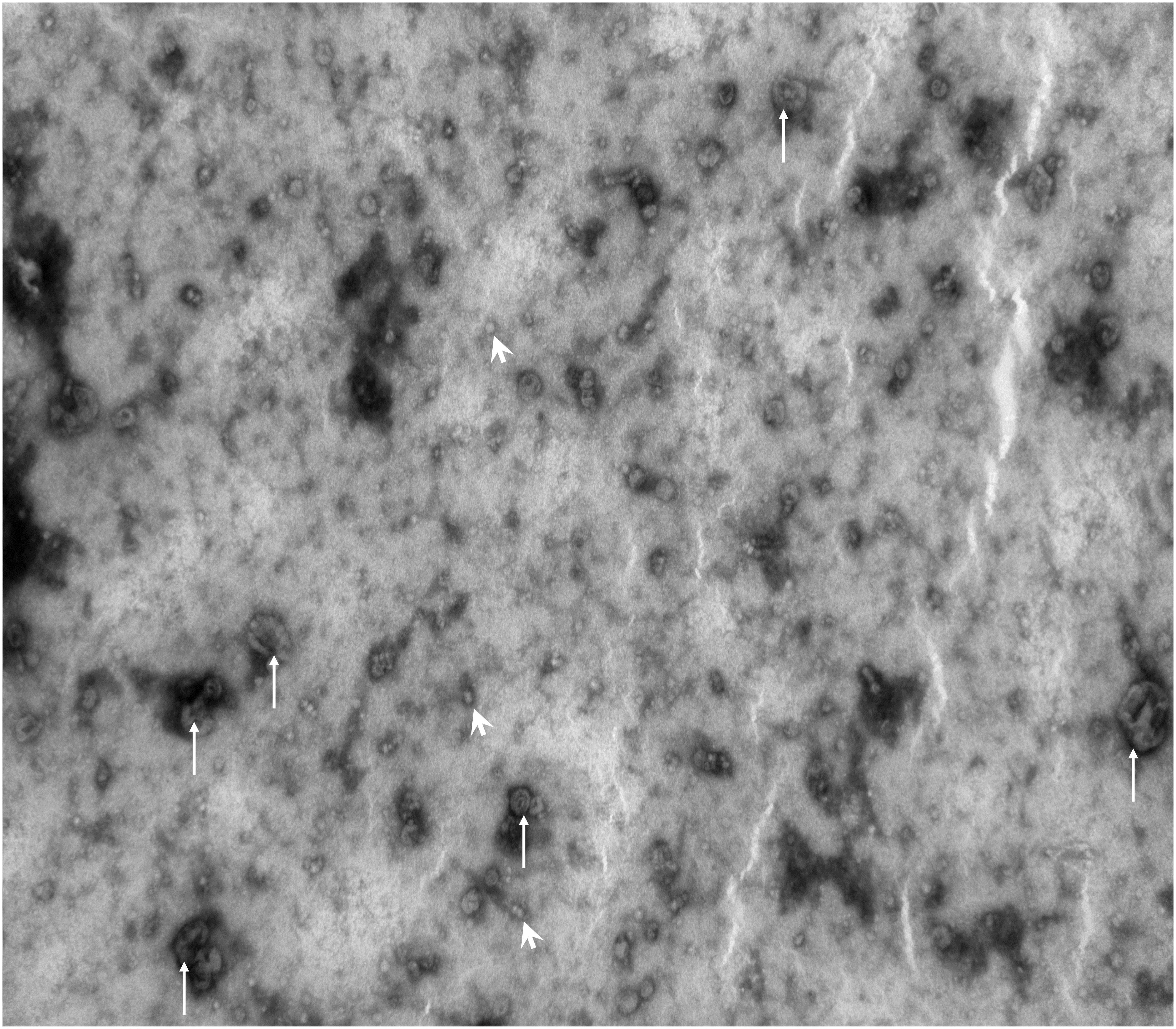

July 20 2012.013.tif
sample 3
Print Mag: 15100x @ 51 mm
16:15 07/20/12

100 nm
HV=80.0kV
Direct Mag: 20000x
AMT Camera System

Supplementary Figure 9c. Heparin-purified EVs

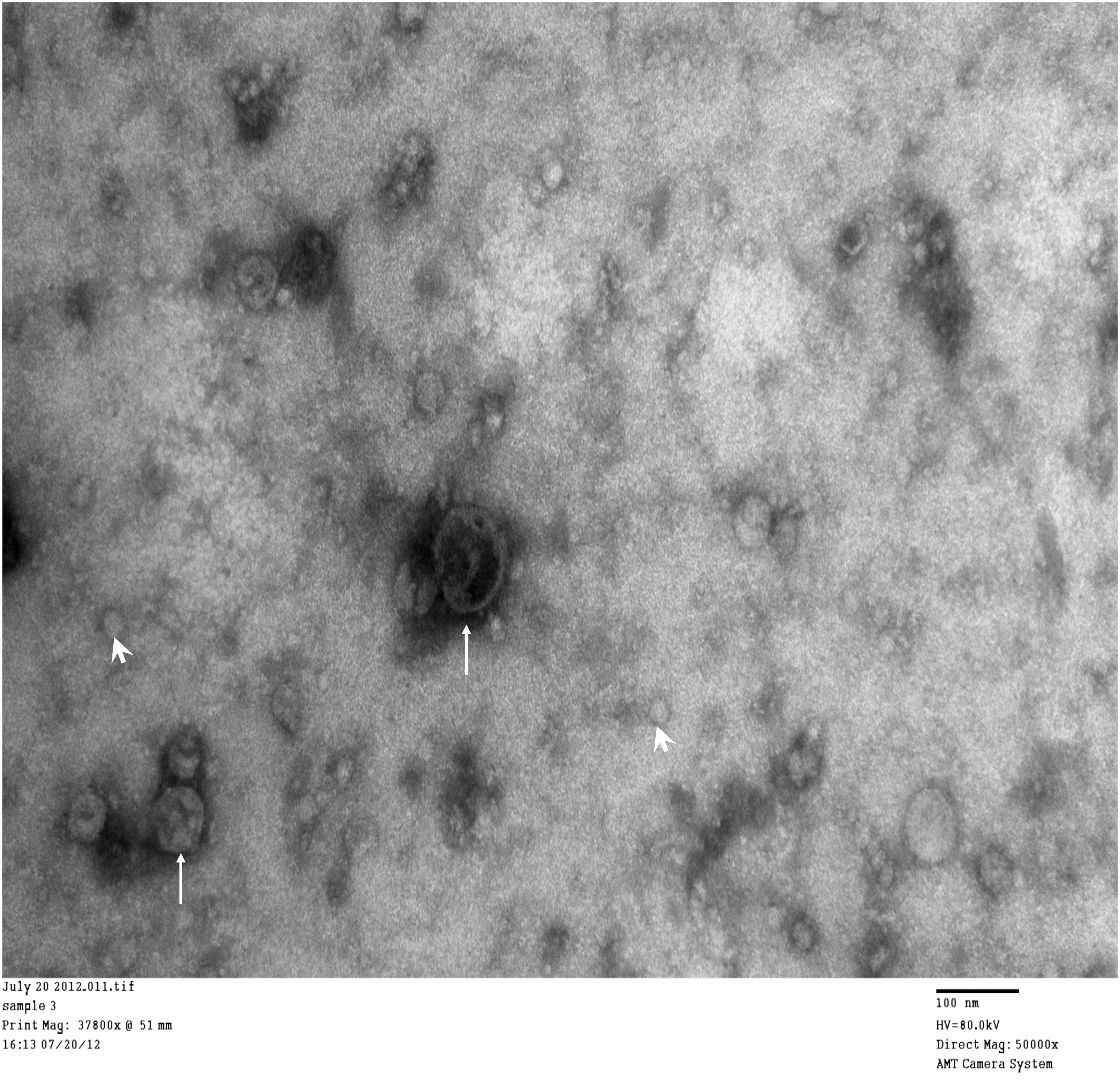

Supplementary Figure 9. TEM of extracellular vesicles isolated using (**a**) ultracentrifugation, (**b**) a commercially available kit, (**c)** heparin coated beads. A low and high magnification image are shown for each sample. Scale bar= 100 nm. Arrows point to large EVs(~50-100 nm) and arrowheads point to small EVs (<~50 nm).
